# ^124^I Radiolabeling of a Au^III^‐NHC Complex for In Vivo Biodistribution Studies†

**DOI:** 10.1002/anie.202008046

**Published:** 2020-07-29

**Authors:** Federica Guarra, Alessio Terenzi, Christine Pirker, Rossana Passannante, Dina Baier, Ennio Zangrando, Vanessa Gómez‐Vallejo, Tarita Biver, Chiara Gabbiani, Walter Berger, Jordi Llop, Luca Salassa

**Affiliations:** ^1^ Department of Chemistry and Industrial Chemistry University of Pisa Via G. Moruzzi 13 56124 Pisa Italy; ^2^ Donostia International Physics Center Paseo M. Lardizabal 4 20018 Donostia Spain; ^3^ Department of Biological, Chemical and Pharmaceutical Sciences and Technologies University of Palermo Viale delle Scienze, Ed. 17 90128 Palermo Italy; ^4^ Department of Medicine I Institute of Cancer Research and Comprehensive Cancer Center Medical University Vienna Borschkegasse 8a 1090 Vienna Austria; ^5^ CIC biomaGUNE Basque Research and Technology Alliance (BRTA) Paseo de Miramón 182 20014 Donostia Spain; ^6^ Institute of Inorganic Chemistry Faculty of Chemistry University of Vienna Waehringerstrasse 42 1090 Vienna Austria; ^7^ Department of Chemical and Pharmaceutical Sciences University of Trieste via Giorgieri 1 34127 Trieste Italy; ^8^ Department of Pharmacy University of Pisa via Bonanno 6 56126 Pisa Italy; ^9^ Kimika Fakultatea Euskal Herriko Unibertsitatea UPV/EHU 20080 Donostia Spain; ^10^ Ikerbasque Basque Foundation for Science 48013 Bilbao Spain

**Keywords:** anticancer, metallodrugs, N-heterocyclic carbenes, positron emission tomography, radiochemistry

## Abstract

Au^III^ complexes with N‐heterocyclic carbene (NHC) ligands have shown remarkable potential as anticancer agents, yet their fate in vivo has not been thoroughly examined and understood. Reported herein is the synthesis of new Au^III^‐NHC complexes by direct oxidation with radioactive [^124^I]I_2_ as a valuable strategy to monitor the in vivo biodistribution of this class of compounds using positron emission tomography (PET). While in vitro analyses provide direct evidence for the importance of Au^III^‐to‐Au^I^ reduction to achieve full anticancer activity, in vivo studies reveal that a fraction of the Au^III^‐NHC prodrug is not immediately reduced after administration but able to reach the major organs before metabolic activation.

## Graphical abstract

**Images**: The reaction of [^124/131^I]I_2_ with Au^I^‐N‐heterocyclic carbene precursors affords a strategy to label Au^III^ anticancer agents and study their biodistribution in vivo by positron emission tomography.

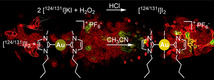

## Introduction

The discovery that auranofin has potent anticancer effects,^1^, ^2^ together with the unceasing quest for metal‐based drug alternatives to cisplatin and its analogues, has stimulated the development of Au complexes as novel chemotherapeutics against cancer.^3^, ^4^, ^5^, ^6^ The pioneering work of Che, Ott, Messori, and Casini, among others, demonstrated that Au^I^ and Au^III^ complexes bearing N‐heterocyclic carbene ligands (NHCs) display unique anticancer activity profiles in vitro^7^, ^8^, ^9^ and reduce tumor size in treated mouse xenografts.^10^, ^11^, ^12^, ^13^, ^14^

While the great majority of the studies have focused on Au^I^‐NHCs, reports on Au^III^ analogues emerged more recently.^15^, ^16^, ^17^, ^18^ Under careful examination, these studies highlight that Au^III^ complexes frequently display antiproliferative effects comparable to their Au^I^ counterparts.^19^, ^20^ The reason for such resemblance likely lies in the limited stability of Au^III^‐NHCs in biological environments and their rapid reduction to Au^I^ metabolites. Surprisingly, this fundamental aspect has been overlooked in a significant portion of the studies reported on Au^III^‐NHCs and their mode of action, generating some confusion on whether Au^III^‐NHCs should solely be considered, and hence designed, as prodrugs of active Au^I^ complexes. Among the exceptions in this panorama is, for example, the work of Che and co‐workers, who prepared a series of Au^III^‐NHCs for intracellular reductive activation by thiols^10^ and demonstrated that the increased stability of pincer cyclometalated Au^III^‐NHCs in biological settings led to remarkable in vitro and in vivo anticancer activities.^11^, ^21^

Furthermore, knowledge concerning the fate of Au^III^‐NHCs after their administration in vivo is still largely insufficient. The study of the biodistribution of Au^III^ compounds in vivo and the evaluation of their metabolization to Au^I^ species is a very challenging task that has not been thoroughly addressed so far, albeit it being a key step in the development of new Au drugs.^22^ Two studies used nuclear imaging and ^198^Au radiolabelling to examine the biodistribution of Au^III^ complexes bearing (bis)thiosemicarbazone‐based and bis(pyrrolide‐imine) Schiff base ligands.^23^, ^24^ Nevertheless, such a radiolabelling strategy is not ideal to investigate the Au^III^‐to‐Au^I^ reduction processes of drug (or prodrug) candidates in animal models, since the radioactive signal of ^198^Au does not report on the changes within the metal coordination sphere and does not provide information on ligand exchange or loss.

In this context, we describe a new strategy that exploits Au coordination chemistry for labeling Au^III^‐NHCs complexes with radioactive ligands and studying their organ accumulation in vivo. In our approach, Au^III^ compounds are labelled with iodine‐124 (^124^I), a radionuclide used for positron emission tomography (PET),^25^ so that they can be unequivocally discerned from their Au^I^ counterparts and tracked by PET. By combining imaging studies with ex vivo analysis, direct evidence of the metabolization and biodistribution of Au^III^‐NHCs overtime can be obtained. Of note, Messori and collaborators recently found that coordination of iodide enhanced the anticancer potency of Au derivatives.^26^

## Results and Discussion

**Figure 1:**
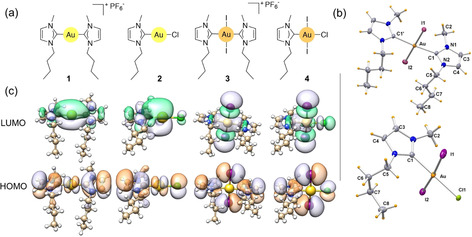
a) Chemical structures of **1**–**4**. b) ORTEP views (ellipsoid probability 50 %) of **3** and **4**. c) DFT‐calculated HOMO and LUMO orbitals for **1**–**4**.

Towards this goal, we selected and prepared Au(I/III) mono‐ and biscarbenic complexes of the 1‐butyl‐3‐methyl‐imidazol‐2‐ylidene NHC ligand (**1**–**4**, Figure 1 a). The Au^I^ complexes **1** and **2**, previously reported by some of us, have potent in vitro antiproliferative activity against cisplatin‐resistant cancer cell lines.^27^, ^28^ Furthermore, **1** showed promising properties both as a thioredoxin reductase inhibitor^29^ and G‐quadruplex binder.^30^ The novel Au^III^ complexes **3** and **4** were obtained by direct oxidation of **1** and **2**, respectively, with elemental iodine. These reactions were carried out in CH_3_CN at room temperature, adapting a procedure reported earlier by Baron et al. for other Au derivatives.^31^ We pursued and achieved a relatively easy‐to‐perform synthesis (see the Supporting Information), with mild experimental conditions and high yields. These are fundamental prerequisites for translating synthetic preparations to the radiochemistry laboratory, where elaborate procedures either need to be minimized or, better, avoided.

The complexes were fully characterized (see the Supporting Information) and X‐ray structures for **3** and **4** were successfully resolved (Figure 1 b; see Figures S1 and S2 and Tables S1 and S2 in the Supporting Information).

To understand the behavior of the Au^III^‐NHCs in biological systems, we evaluated their stability in solution by UV/Vis and ^1^H NMR spectroscopy. Au^III^‐to‐Au^I^ reduction was monitored by either the decrease of the characteristic Au^III^ absorption band between 300–450 nm (see Figure S3) or by the shift of the ^1^H NMR peak relative to the NHC ligands at around *δ*=7 ppm (see Figure S4). Because of the presence of *syn*/*anti* isomers (see Figure S5), **3** showed a double set of ^1^H NMR signals for the alkyl chains of the NHC ligands.

UV/Vis and ^1^H NMR spectroscopy showed that **3** was stable in phosphate buffer (PB) and phosphate buffer saline (PBS) over 24 hours (see Figures S6 and S7). Instead, the UV/Vis spectra of **4** changed significantly in the same time window (see Figure S8) and ^1^H NMR spectroscopy in PBS indicated that the monocarbene evolved to form **3** (see Figure S9). Furthermore, **3** was stable for approximately 30–60 minutes in a cell culture medium containing fetal bovine serum (see Figure S10 and S11).

DFT analysis of **3** and **4** revealed that they had LUMO orbitals with σ‐antibonding character toward the Au−I bonds, in agreement with their tendency to undergo reductive elimination reactions. Conversely, **1** and **2** displayed bonding LUMOs toward Au‐NHC bonds (Figure 1 c), possibly accounting for the good stability of Au^I^‐NHCs in biological environments.^28^ Similar results were found for the *anti* isomers of **3** and **1** (see Figure S12). Overall, experiments and theory confirmed that **3** had superior stability relative to **4**. For this reason, we selected **3** for the radiochemistry and in vivo work.

Before in vivo tests, we conducted in vitro studies comparing **1** and **3** to examine the impact of the Au^III^/Au^I^ reduction process on the anticancer effects. A viability screening of different cancer cell lines, comprising models with acquired resistance against cisplatin and oxaliplatin, was performed. The activities of **1** and **3** were also compared to auranofin (Table 1). While both Au‐NHCs showed strong antiproliferative activities, with IC_50_ values falling in the low micromolar range, only moderate differences between **1** and **3** were observed. For instance, **3** was more active than **1** in some of the p53 wild‐type models (HCT116‐p53wt, A2780). Moreover, knock‐out of p53 significantly sensitized cells against **1** but not against **3**, confirming the impact of p53 signaling on either uptake or activation of the two Au‐NHCs, as recently reported for **1**.^27^

**Table 1:** Growth inhibition of different cancer cell lines after a 72 hour drug exposure.

Cell lines	**1**	**3**	Auranofin	Oxaliplatin
HCT116‐p53wt	4.0±0.3	2.0±0.1	3.7±0.1	0.8±0.1
HCT116‐p53wt/OxR	1.4±0.6	1.3±0.5	2.4±0.5	>10
HCT116‐p53ko	1.8±0.1	2.4±0.7	3.0±0.4	2.6±0.1
HCT116‐p53ko/OxR	>10	>10	3.51±0.3	>10
A2780	2.0±0.4	1.8±0.6	0.9±0.2	–
A2780cis	1.0±0.5	0.9±0.6	3.1±0.4	–
MCF‐7	1.5±0.5	2.7±0.1	2.2±0.4	–
A375	1.7±0.4	1.8±0.5	1.4±0.4	3.8±0.3
N87	5.3±2.4	6.2±4.7	6.1±4.6	–

An analysis of the Pt resistance cell models revealed a clear dissimilarity in the mode‐of‐action and/or resistance behavior of the two Au‐NHCs compared to auranofin. For example, the cisplatin‐resistant subline of A2780 exhibited hypersensitivity against **1** and **3** compared to the parental cell line, while, in sharp contrast, resistance against auranofin was observed. The situation was reversed in the HCT116‐p53ko background with acquired oxaliplatin resistance, which induced only a moderate insensitivity against auranofin but a marked one against both **1** and **3**.

Hoechst/PI staining highlighted that auranofin predominantly induced cell death (PI‐positive red cells), while **1** and **3** were predominantly cytostatic (see Figure S13). Accordingly, cell cycle distribution analysis revealed that a 24 hour incubation with both **1** and **3** induced a dose‐dependent cell accumulation in the G1 phase of different models, with the exception of the A2780 parental cells (see Figure S14). Furthermore, an ATP‐based assay indicated for both Au‐NHCs (and to a much lesser extent for auranofin) a higher activity compared to the MTT‐based results (see Figure S15), suggesting a strong effect of Au‐NHC compounds on mitochondrial ATP production. Viability assays in the A2780 and HCT116 models were also performed in the presence of *N*‐acetyl cysteine (NAC), a known reactive oxygen species (ROS) scavenger and reducing agent. While the anticancer activities of **1** and **3** remained widely unaffected, we observed a clear protective effect for auranofin in all drug‐sensitive parental models (see Figure S16).

The sum of these data suggested a different mode‐of‐action for the Au‐NHCs as compared to auranofin. However, an overall similar toxicity profile was observed for **1** and **3**, advocating that the reduction of **3** to yield **1** took place extremely rapidly in the cell culture medium. This reduction could occur even before addition to the cell cultures, considering that viability experiments required initial dilutions of the compounds in the cell culture medium supplemented with FBS (in which **3** is poorly stable as shown in Figure S11).

**Figure 2:**
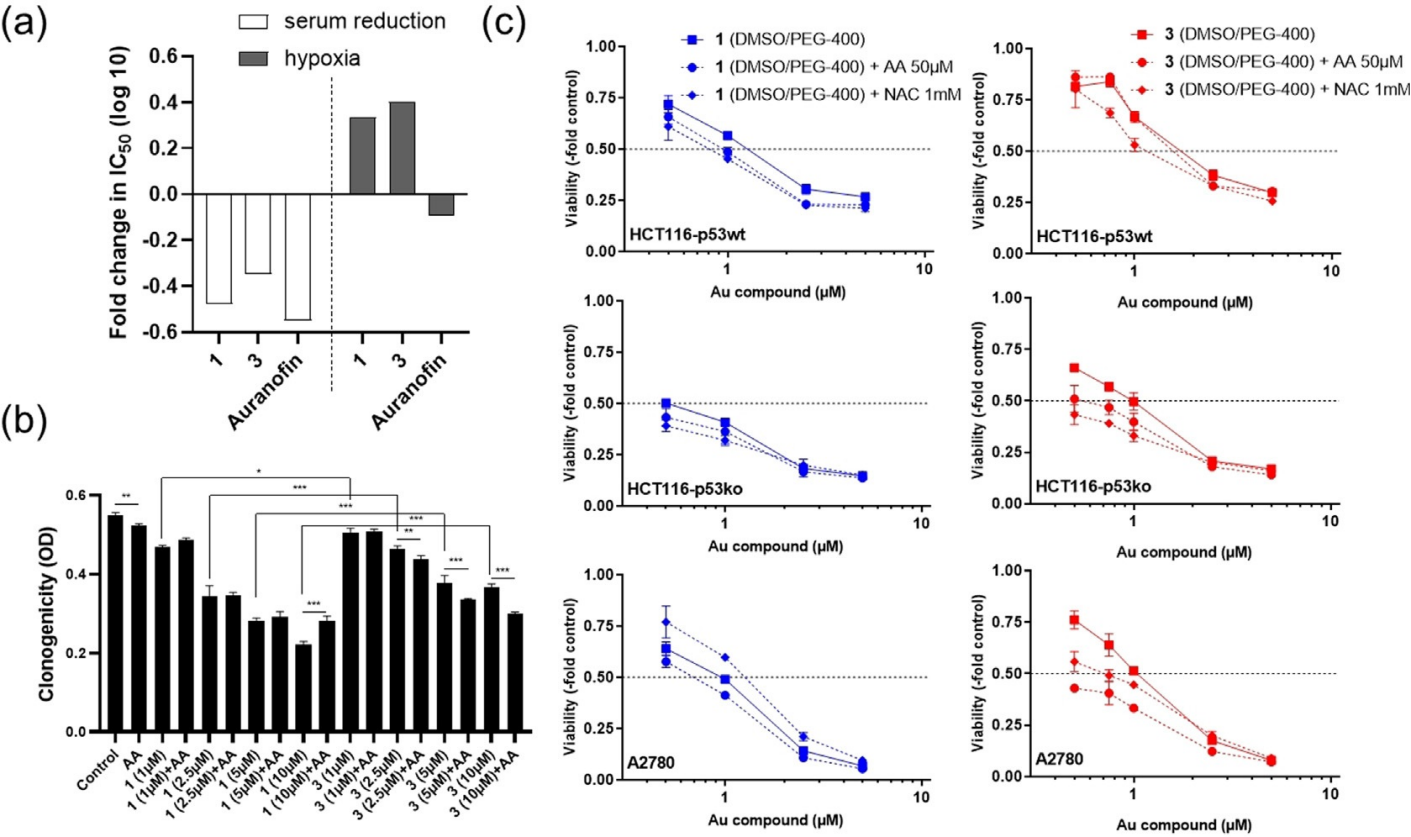
Impact of cell culture conditions on the anticancer activity of Au‐NHC compounds. a) Impact of serum reduction (0.1 % vs. 10 %) and hypoxia (1 % oxygen) on the IC_50_ values of the indicated drugs. b) Impact of Au‐NHCs on the clonogenicity of A2780 cells, pre‐incubated for 2 h in PBS with either **1** or **3** without/with AA (50 μm). Mean values and standard deviations (SDs) of at least two experiments in triplicate. c) Impact of co‐incubation with reducing agents (AA, 50 μm; NAC 1 mm) on the anticancer activity of **1** (blue, left) or **3** (red, right) prepared as the PEG‐400‐based formulation. Mean values and SDs of representative experiments out of at least two performed in triplicate.

Therefore, we performed MTT assays either in the absence of serum or under hypoxic conditions (Figure 2 a). Serum reduction to 0.1 % resulted in an enhanced activity for all Au complexes, whereas the reducing hypoxic environment sensitized against auranofin but protected against Au‐NHCs.

The comparable response of **1** and **3** to these specific experimental conditions highlighted that the Au^III^ reduction process was not critical and other factors played a role (e.g. inactivation of the Au complexes by a serum component). For this reason, we performed another experiment, exposing A2780 cells to the Au complexes for 2 hours in PBS with and without ascorbic acid (AA) as a reducing agent, followed by a colony formation assay without drugs in 24‐well plates. The densitometric evaluation revealed an enhanced activity of **1** compared to **3** over the entire concentration range, as well as a significant chemosensitization of **3** by AA (Figure 2 b). On the contrary, **1** tended to be even less active in the presence of AA, ultimately proving that **3** needs reduction for exhibiting optimal anticancer activity in vitro.

To mimic the in vivo sample administration conditions, we used, in addition to the standard DMSO stock solution, also a PEG‐400‐based formulation (30 % PEG, 5 % DMSO, 65 % PB) of the two Au‐NHCs in vitro. Mostly, the IC_50_ values for the PEG‐400 formulation remained in a comparable concentration range, but the activity of **1** tended to be increased whereas **3** tended to be less active in several cell models (violin blot in Figure S17a). As an illustrative example, selected dose‐response curves for MCF‐7 breast cancer cells are shown in Figure S17b. These data suggest that the reduction process of **3** into **1** might be delayed by the presence of PEG‐400, depending on the cellular background. Additionally, the PEG‐400 formulation at concentrations above the IC_50_ value was more active than the DMSO counterpart, suggesting upregulation of the cytotoxic activities of both **1** and **3** (HCT116 cell models shown representatively in Figure S17c). Compared to auranofin, **1** and **3** induced a stronger upregulation of the pro‐apoptotic bax protein involved in auranofin‐mediated apoptosis induction (Figures S17d).^32^ Despite evidence of PARP cleavage by all Au compounds, this result clearly indicated a different interaction with cell death regulatory pathways for auranofin and AuNHCs. The formulation effect was able to overcome the resistance against the Au‐NHCs observed in the oxaliplatin‐resistant HCT116‐p53ko/OxR model (compare Figure S17c). To prove whether **3** was protected against immediate reduction to **1** in the presence of PEG‐400, combination experiments with AA and NAC were performed. Indeed, a sensitizing effect of coincubation with the two reducing agents was primarily observed in the case of **3** while the effects were variable and minor for **1** (Figure 2 c). Together, the PEG‐400 formulation had superior performance in terms of anticancer activity, resistance circumvention, and, especially, delayed prodrug activation essential for in vivo biodistribution studies.

**Figure 3:**
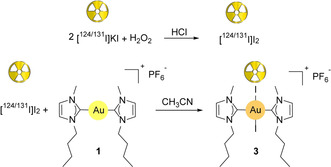
Scheme for the radiochemical synthesis of **3**.

Radiolabeling of **3** for in vivo work was carried out following the scheme of Figure 3. Longer lived (and less expensive) ^131^I (*t*_1/2_=8.02 days) was used for optimizing the reaction conditions in the radiochemistry laboratory and for time–activity studies in blood, while the radionuclide ^124^I (*t*_1/2_=4.18 days) was employed in the sample preparation for the in vivo PET imaging experiments. In brief, a water solution containing KI spiked with [^124/131^I]KI was oxidized with H_2_O_2_ under acidic conditions to obtain elemental radioactive iodine by precipitation. The solid was then isolated by centrifugation, washed with water and reacted with **1** in CH_3_CN for 1 hour. As determined by HPLC connected in series with a radioactivity detector, we obtained a 95 % purity of the product (see Figure S18). The radiochemical yield (35 %) was relatively high considering a maximum theoretical yield of 40 % (see the Supporting Information).

Because of its modest water solubility (<0.4 mg mL^−1^, 15 % DMSO) and the toxicity of DMSO at high concentrations, radiolabeled **3** was solubilized in a PEG‐400 based formulation (30 % PEG, 5 % DMSO, 65 % PB) for subsequent intravenous injection in rodents. We selected such a formulation on the basis of tolerability and solvent dose limits suggested by Thackaberry et al.^33^ Indeed, [^124^I]KI dissolved in this mixture and when used as a control proved to be well tolerated by intravenously administered rats. HPLC experiments confirmed that radiolabeled **3** was stable over 24 hours in the PEG‐400 formulation (see Figure S19). Crucially, time‐dependent UV/Vis spectra indicated that formulated **3** was more stable than the complex alone in PBS against reduction by stoichiometric amounts of AA (Figure S20). In buffer, **3** was completely reduced by AA in less than 20 minutes, whereas in the PEG formulation the Au^III^‐associated UV/Vis band at 300–450 nm could be observed even after 2 hours (ca. 70 % reduction).

**Figure 4:**
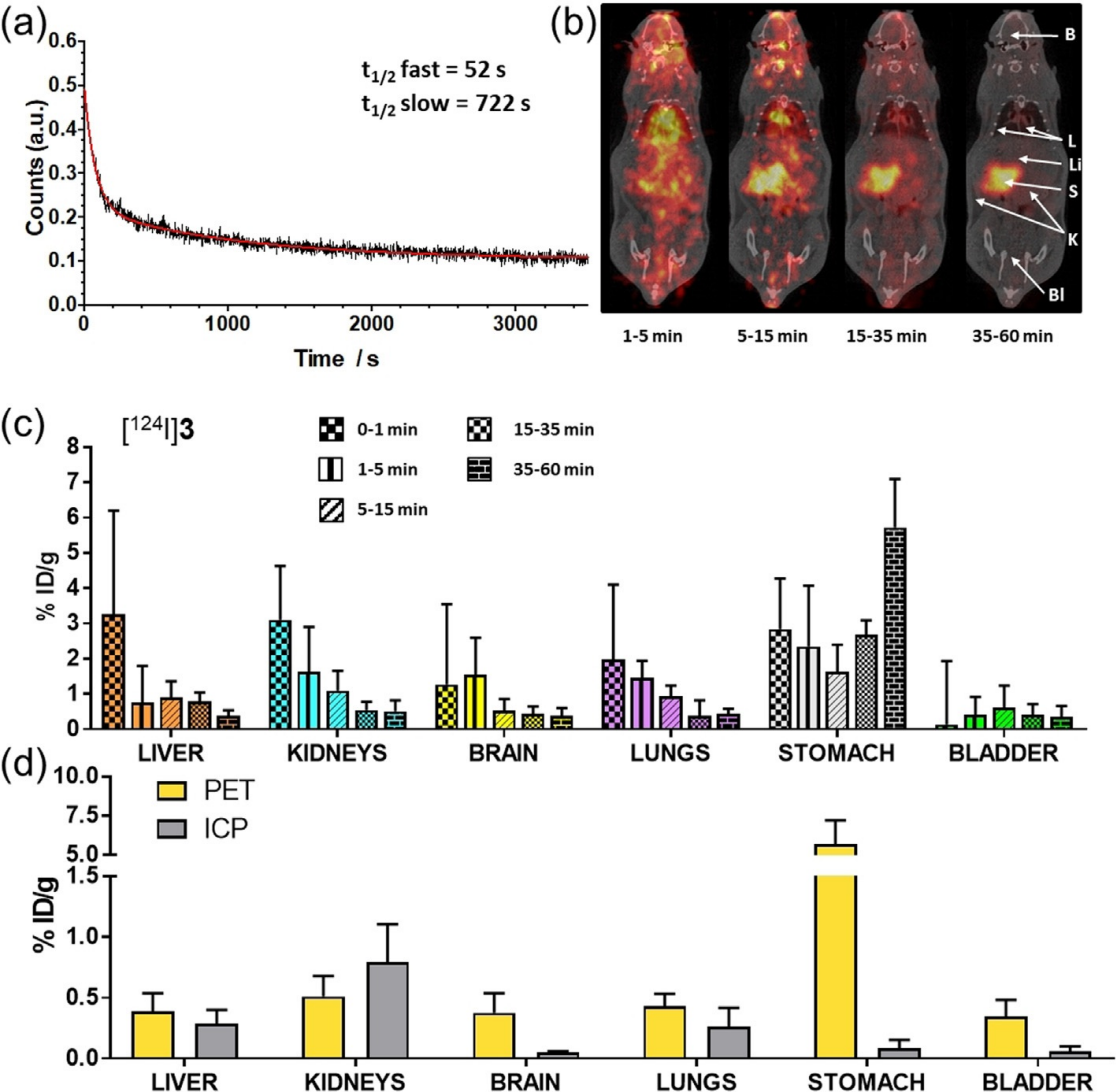
a) Time‐activity curve of arterial blood as determined by on‐line blood sampling after intravenous administration of [^131^I]**3**. In red, the biexponential curve fitted to the experimental data. b) PET images (maximum intensity projections) obtained at different time intervals after intravenous administration of [^124^I]**3**. PET images have been co‐registered with representative computer tomography (CT) slices for localization of the radioactive signal. c) Concentration of radioactivity in different organs and at different times after administration of [^124^I]**3**, as determined from PET images. Histograms are obtained by averaging the %ID g^−1^ values for the indicated time frame (ID: injected dose). d) Concentration of radioactivity (as determined by PET) and concentration of Au (as determined by ICP‐MS) in different organs.

In a first in vivo experiment, we investigated the biological half‐life of **3**, measuring its time‐activity curve in blood (that is, the concentration of radioactivity in blood as a function of time). Towards this goal, the arterial input function was determined by catheterization and online blood sampling using a previously reported procedure with minor modifications.^34^ In brief, a 640 μm solution of [^131^I]**3** dissolved in the PEG‐400 formulation (1.11 MBq) was intravenously administered to a wild‐type rat bearing a bypass between the femoral artery and the femoral vein to achieve an extracorporeal circulation of the blood through a radioactivity detector. Blood concentration of the radioactive signal versus time was measured and the resulting curve was analyzed using a nonlinear regression method (Figure 4 a).

The pharmacokinetic profile of [^131^I]**3** fitted well with a biexponential equation indicating a fast distribution phase (*t*≈1 min) followed by a slower clearance phase (*t*≈12 min). In the same experiment, blood samples were collected at different time points, and analyzed by HPLC to assess the fraction of [^131^I]**3** that remained unaltered. Already after 4 minutes, no radioactive peak at retention time (rt)=8–8.5 min could be observed, confirming the absence of [^131^I]**3** in the blood. Only one peak was observed in the chromatogram (rt=2.2 min; see Figure S21), corresponding to a radioactive degradation product, most probably free iodide. The same peak was also observed in the radio‐chromatogram at longer time points after injection (results not shown).

Subsequently, we explored the accumulation of radioactivity in the whole body after intravenous administration of [^124^I]**3** (1.2 MBq, 640 μm) using PET imaging (Figure 4 b). The amount of compound injected per animal was about 0.45 mg kg^−1^, which is in the range of pharmacological doses used in therapeutic studies. Hence, no further dilution with the nonradioactive compound was carried out. [^124^I]KI (1.9 MBq, 1 mm) was also administered in a separate animal (see Figure S21a) using the PEG‐400 formulation as a control. Visual inspection of PET images (Figure 4 b) showed high accumulation of radioactivity in the heart at short times after administration (*t*=1–5 min). During this time frame, high levels of radioactivity were clearly observed in the liver, kidneys, lungs, brain, and the stomach. Except for the latter, quantification of the PET images (Figures 4 c) indicated that these initial values progressively decreased with time to reach negligible values after 35–60 minutes. These results suggested that [^124^I]**3** was rapidly distributed in the blood pool and localized in these organs, then reduced with the overtime loss of iodido ligands [^124^I]I^−^, which are the species producing the radioactive signals. Indeed, whereas poor accumulation was observed in the bladder, radioactivity values in the stomach remained high throughout the PET study, reaching approximately 6 % ID cm^−3^ (ID: injected dose) at the last time point. Such accumulation was expected since the stomach is the natural site of iodine metabolism.^35^

Additionally, we determined the body distribution of Au by ICP‐MS harvesting the organs of the rats immediately after termination of the imaging sessions. Comparing such data with the PET results in terms of %ID per gram of tissue (Figure 4 d), we observed a good correlation in the liver, kidneys, and lungs, confirming the rapid accumulation of **3** in such organs. At the same time, the differences between ICP‐MS and PET quantification were visible in the bladder and the brain, and were particularly significant in the stomach. This data clearly indicated that the Au concentration was low and that free [^124^I]I^−^ was what we primarily observed in the images of these tissues.

A comparative analysis with the PET scan of the control [^124^I]KI (see Figure S21) provided further insights on the in vivo fate of **3**. The accumulation profile of [^124^I]KI in the lungs, kidneys and liver resembled the one observed for [^124^I]**3**. However, [^124^I]KI prevalently accumulated in the bladder with values close to 5 % ID cm^−3^ at *t*=15–60 minutes post‐administration, and to some extent also in the stomach, although significantly less than in the case of [^124^I]**3**. These important differences in the biodistribution of [^124^I]**3** and [^124^I]KI indicated that the in vivo reduction of **3** and subsequent release of iodide, although rapid, was not instantaneous, in agreement with its improved stability in the PEG‐400 formulation. Within the duration of the PET study, the complex, or at least a significant fraction of it, reached the major organs (i.e. lungs, kidneys and liver) where it accumulated and underwent further metabolization as demonstrated by comparing PET and ICP‐MS data.

## Conclusion

In summary, we reported the synthesis and characterization of new mono‐ and bis Au^III^‐NHCs, including their X‐ray structures. In vitro studies revealed a fast Au^III^‐to‐Au^I^ reduction mechanism. Nevertheless, specific cell culture conditions (i.e. avoiding serum in the incubation protocol) or the use of a PEG‐400 formulation promoted a lower activity of **3** with respect to **1**, as well as activation of **3**, but not **1**, in the presence of reducing agents. Importantly, PEG‐400 formulation improved the performance of **3** in terms of anticancer activity and resistance circumvention. Such results suggested that fast reduction of **3** to **1** was needed for triggering of the full anticancer activity of the Au^III^‐NHC derivative, indicating that **3** acts as a prodrug of **1**. Additionally, contrary to what reported in the literature for **1** and other similar derivatives,^27^, ^36^ the mode‐of‐action profile of **3** and **1** strikingly differs from that of auranofin, the reference Au compound to date. The different interference with platinum drug resistance mechanisms represents here one illustrative example. The biscarbene **3** was labeled for in vivo PET imaging by direct oxidation with radioactive [^124/131^I]I_2_. This novel approach enabled dissecting the reduction path of this compound once intravenously injected into healthy rats. The complex **3** was rapidly, but not immediately, reduced to its Au^I^ analogue as captured by PET and ICP in comparison with the [^124^I]KI control. In conclusion, the present study, while urging the medicinal inorganic chemistry community to examine in detail the metabolization of Au^III^ compounds, highlights the potential of our radiolabeling strategy with ^124^I to unravel the fate of Au^III^‐NHC drug candidates in vivo. Crucially, the same strategy could be applied to study the in vivo biodistribution of other metal‐based drugs, including Pt^IV^ prodrugs which are currently in different clinical trials.

## Conflict of interest

The authors declare no conflict of interest.

## Supporting information

As a service to our authors and readers, this journal provides supporting information supplied by the authors. Such materials are peer reviewed and may be re‐organized for online delivery, but are not copy‐edited or typeset. Technical support issues arising from supporting information (other than missing files) should be addressed to the authors.

Supplementary
