# Supplementary material for: 124I Radiolabeling of a AuIII‐NHC Complex for In Vivo Biodistribution Studies
Source: Angew Chem Int Ed Engl. 2020 Jul 29;59(39):17130–6. doi: 10.1002/anie.202008046 (PMC7540067; doi:10.1002/anie.202008046)
Supplement: Supplementary file 1 — Supplementary [file ANIE-59-17130-s001.pdf]

Angewandte Chemie
Eine Zeitschrift der Gesellschaft Deutscher Chemiker

Supporting Information

^{124}I Radiolabeling of a Au^{III}-NHC Complex for In Vivo Biodistribution Studies**

Federica Guarra[+], Alessio Terenzi[+], Christine Pirker, Rossana Passannante, Dina Baier, Ennio Zangrando, Vanessa Gómez-Vallejo, Tarita Biver, Chiara Gabbiani, Walter Berger,* Jordi Llop,* and Luca Salassa**

anie_202008046_sm_miscellaneous_information.pdf

Author Contributions

F.G. Conceptualization: Equal; Data curation: Equal; Formal analysis: Equal; Investigation: Equal; Writing—Original Draft: Equal
A.T. Conceptualization: Lead; Formal analysis: Lead; Funding acquisition: Lead; Investigation: Lead; Methodology: Lead; Supervision: Lead; Writing—Original Draft: Lead; Writing—Review & Editing: Lead
C.P. Formal analysis: Equal; Investigation: Equal; Methodology: Supporting
R.P. Investigation: Supporting; Methodology: Supporting
D.B. Investigation: Supporting
E.Z. Investigation: Supporting; Methodology: Supporting; Writing—Original Draft: Supporting
V.G. Investigation: Supporting; Methodology: Supporting; Supervision: Supporting
T.B. Conceptualization: Supporting; Supervision: Supporting; Writing—Original Draft: Supporting
C.G. Conceptualization: Equal; Supervision: Supporting; Writing—Original Draft: Supporting
W.B. Formal analysis: Lead; Methodology: Lead; Supervision: Lead; Writing—Original Draft: Lead; Writing—Review & Editing: Lead
J.L. Conceptualization: Lead; Data curation: Lead; Formal analysis: Lead; Investigation: Lead; Methodology: Lead; Writing—Original Draft: Lead; Writing - Review & Editing: Lead
L.S. Conceptualization: Lead; Funding acquisition: Lead; Investigation: Lead; Methodology: Lead; Supervision: Lead; Writing—Original Draft: Lead; Writing—Review & Editing: Lead.

Supplementary Information

Supplementary Tables

Table S1. Crystallographic Data and Details of Refinements for complexes **3** and **4**.

	**3**	**4**
empirical formula	$C_{16}H_{28}AuF_6I_2N_4P$	$C_8H_{14}AuClI_2N_2$
fw	872.16	624.43
cryst system	Orthorhombic	Monoclinic
space group	*P* nma	*P* 2_1/n
a (Å)	13.952(3)	8.596(2)
b (Å)	12.454(2)	13.448(3)
c (Å)	14.719(3)	12.118(2)
β (°)	90.0	95.64(3)
V (Å^3)	2557.5(9)	1394.0(5)
Z	4	4
D_{calcd} (mg/m^3)	2.265	2.975
μ (Mo-Kα) (mm^{-1})	8.283	15.144
F(000)	1624	1112
θ range (°)	1.98 - 28.22	2.23 - 28.22
collected reflections	40468	43749
indep reflections	3421	3564
*R*int	0.0398	0.0491
Obs reflcs [*I* > 2σ(*I*)]	3395	3526
parameters	148	130
*R*1 [*I* > 2σ(*I*)] [a]	0.0248	0.0341
*wR*2 [*I* > 2σ(*I*)] [a]	0.0643	0.1033
GOF on F^2	1.103	1.085
residuals (e Å^{-3}) [b]	1.840, -1.646	2.521, -3.457

[a] $R1 = \Sigma \,||Fo| - |Fc||\, / \Sigma |Fo|$, $wR2 = [\Sigma\, w\, (Fo^{\,2} - Fc^{\,2})^{\,2} / \Sigma w\, (Fo^{\,2})^{\,2}\,]^{½}$
[b] close to the metal center

Table S2. Coordination bond distances (Å) and angles (°) of complexes **3** and **4**.

**3**			
Au-C(1)	2.037(4)	Au-I(2)	2.6326(9)
Au-I(1)	2.5872(8)		
C(1')-Au-C(1)	175.62(19)	C(1)-Au-I(1)	88.40(10)
I(1)-Au-I(2)	179.690(12)	C(1)-Au-I(2)	91.61(10)
C(1') at x,-y+3/2,z			
**4**			
Au-C(1)	2.004(6)	Au-I(1)	2.5976(8)
Au-Cl(1)	2.3445(14)	Au-I(2)	2.6230(8)
C(1)-Au-Cl(1)	178.54(15)	C(1)-Au-I(2)	89.20(15)
I(1)-Au-I(2)	172.941(17)	Cl(1)-Au-I(1)	93.48(4)
C(1)-Au-I(1)	85.17(15)	Cl(1)-Au-I(2)	92.18(4)

Supplementary Figures

X-ray structural analysis. X-ray quality crystals of complexes **3** and **4** were successfully obtained by liquid-liquid diffusion. Diffraction data were collected at the X-ray diffraction beamline (XRD1) of the Elettra Synchrotron (Trieste, Italy), at 100 K with a monochromatic wavelength of 0.700 Å The structural analysis revealed that complex **3** comprises an Au(III) metal center oriented in a rigorously square-planar environment with two trans-carbene ligands and two trans-iodide ions, counterbalanced by a PF_6^- anion. The two imidazole rings are almost coplanar (dihedral angle between their mean planes = 4.6°), while they are twisted by 84.69° relative to the C_2I_2 coordination plane. The neutral complex **4** shows a structure comparable to that of **3**, having one carbene ligand replaced by a chloride. Bond distances and angles of both compounds (see Table S2) are comparable to those measured for structurally related Au(III) complexes.[1–3]

CCDC 1989306-1989307 contain the supplementary crystallographic data for this paper. These data are provided free of charge by The Cambridge Crystallographic Data Centre via www.ccdc.cam.ac.uk/data_request/cif, or by application to the Director, CCDC, 12 Union Road, Cambridge CB21EZ, UK (fax: Int. code +(1223)336-033; e-mail: deposit@ccdc.cam.ac.uk).

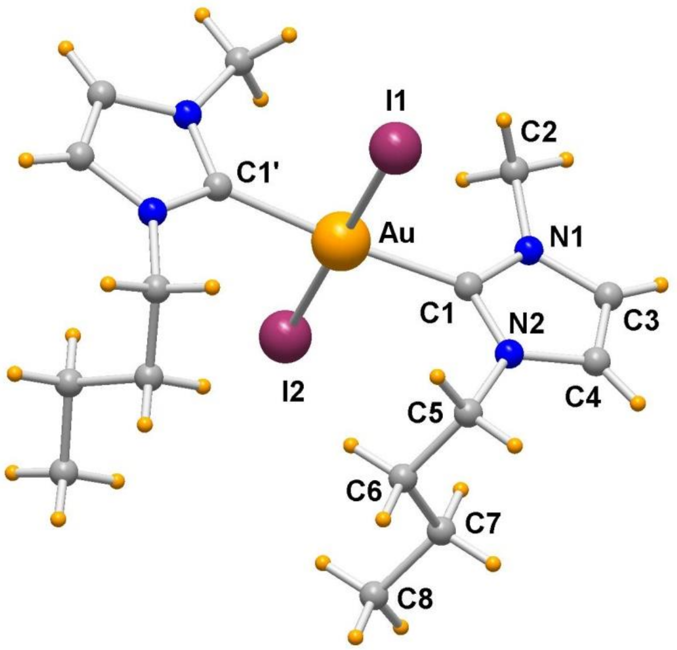

Figure S1. Molecular structure (ORTEP drawing, ellipsoid probability at 50%) of complex cation of **3** located on a crystallographic symmetry mirror including gold and iodine atoms. Primed atom at x, -y+3/2, z.

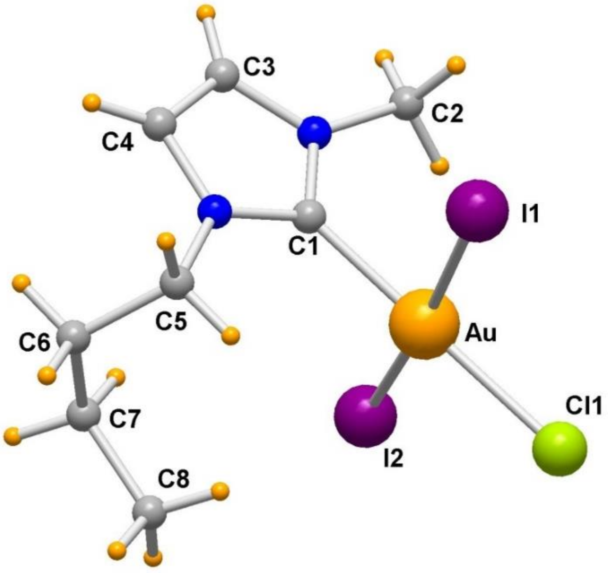

Figure S2. Molecular structure (ORTEP drawing, ellipsoid probability at 50%) of complex **4**.

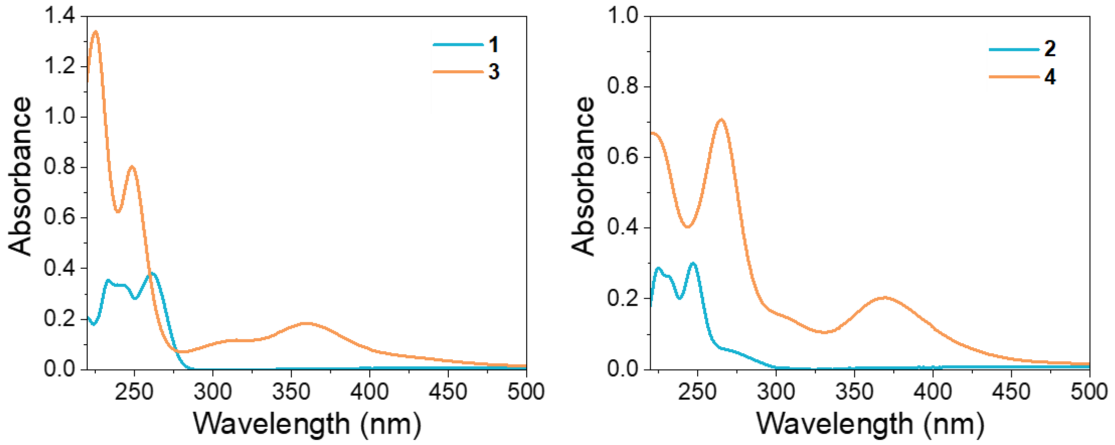

Figure S3. UV-Vis spectra in PB (50 mM, pH = 7.4) with 0.6% CH_3CN of compounds 30 µM **3** (left panel) and **4** (right panel) compared to the corresponding spectra of their reduced form, **1** and **2** respectively.

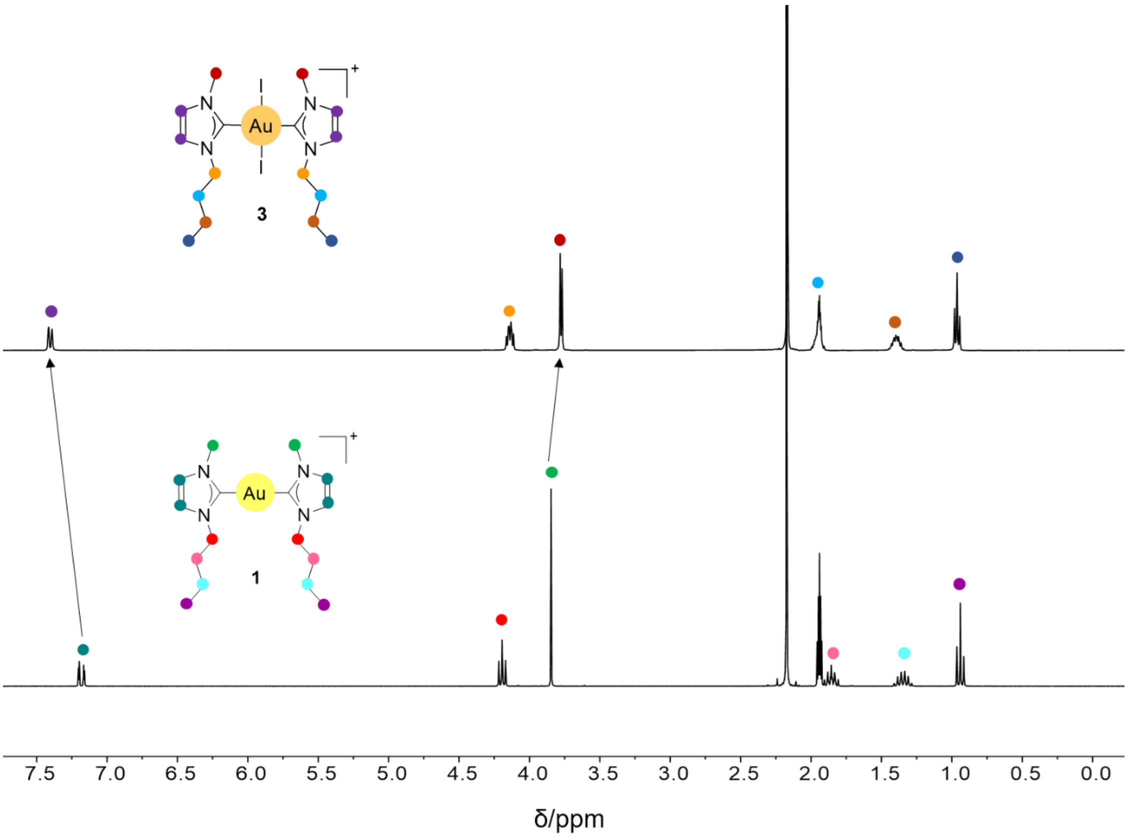

Figure S4. Stacked ^{1}H NMR spectra of compounds **1** and **3** in CD_3CN with peak assignment**.**

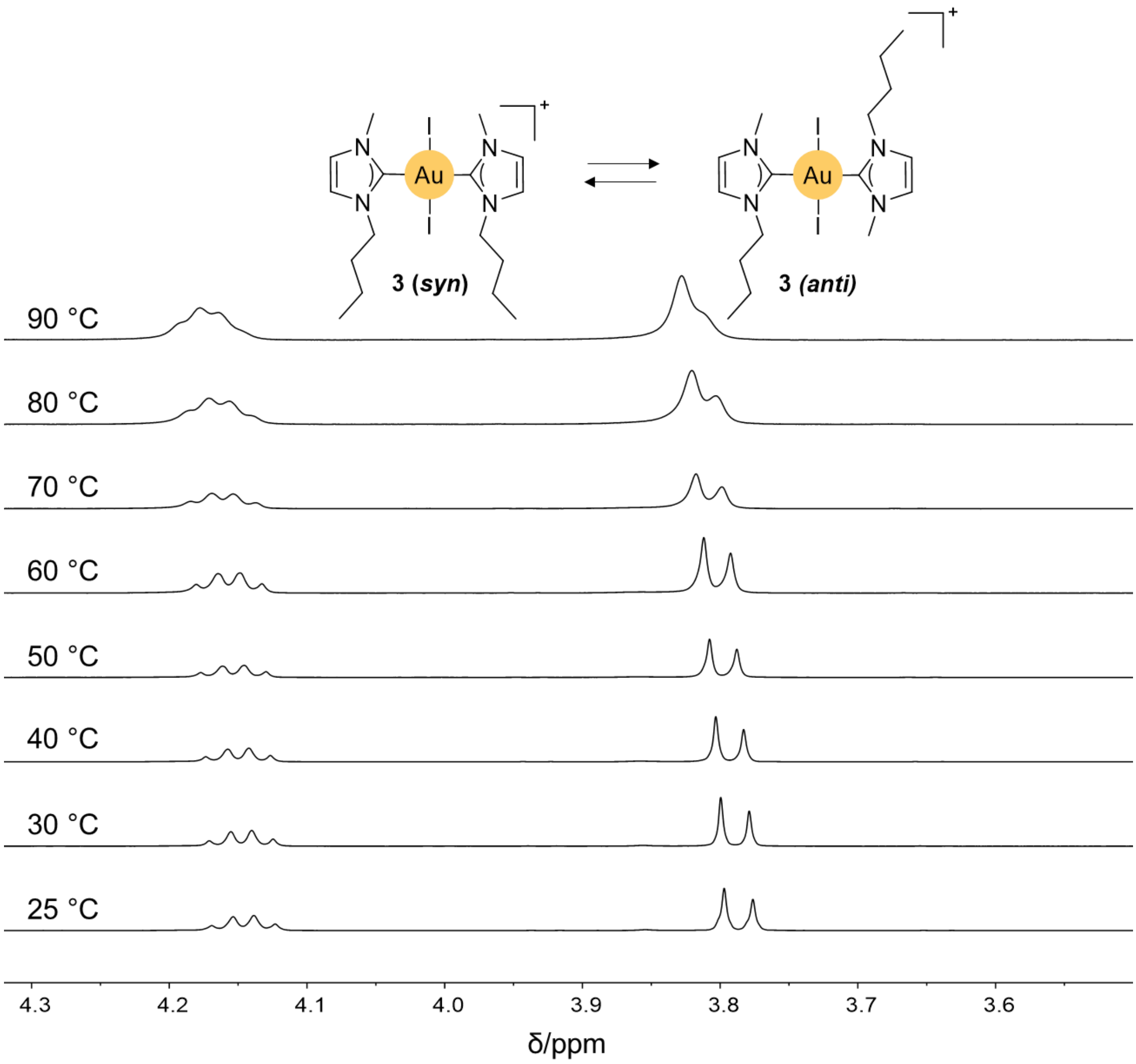

Figure S5. Temperature dependent ^{1}H NMR spectra of compound **3** in d_6-DMSO to show the the presence of *syn*/*anti* isomers. The double set of ^{1}H-NMR signals for the alkyl chains of the NHC ligands tends to disappear when the temperature is increased.

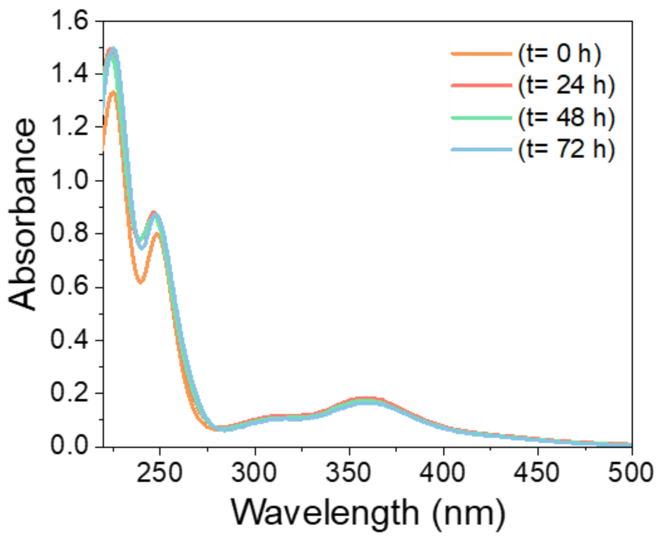

Figure S6. Time dependent UV-Vis spectra of 30µM **3** in PB (50 mM) with 0.6 % CH_3CN.

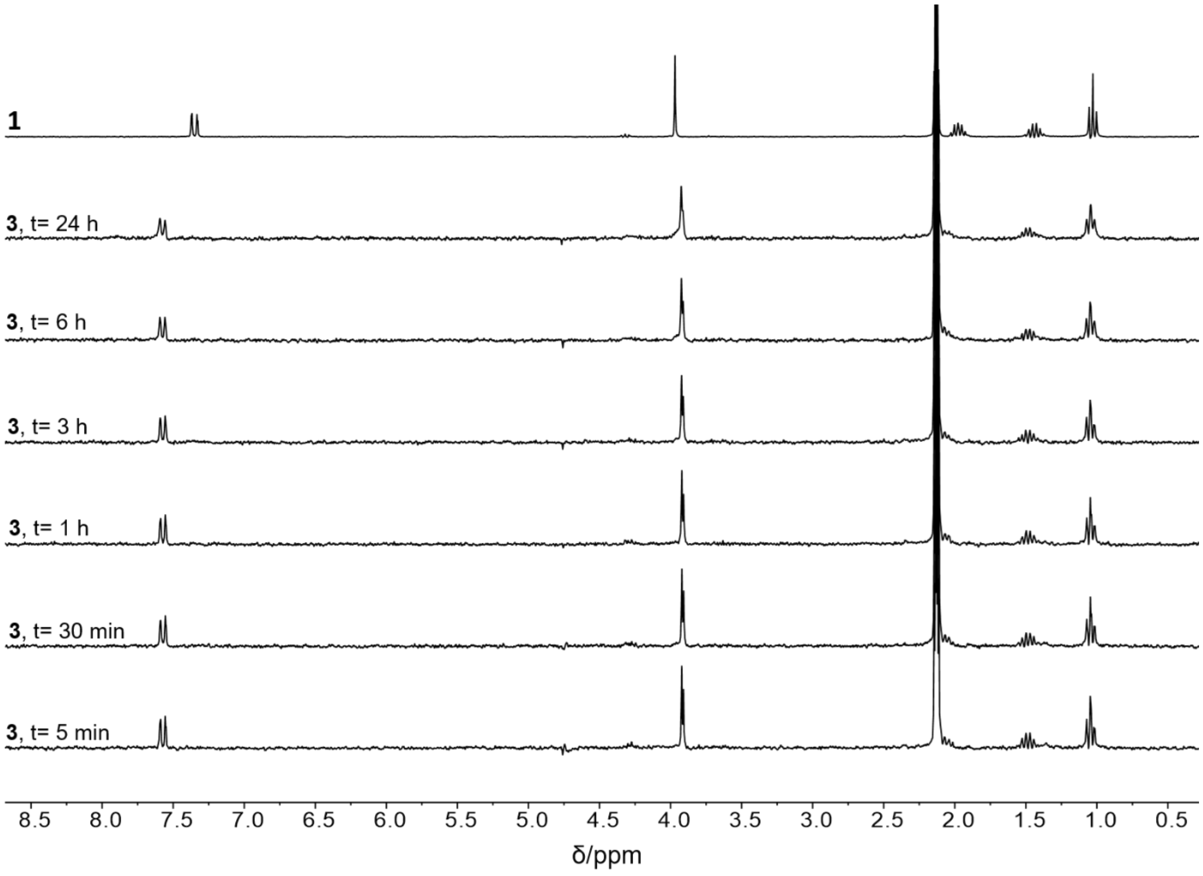

Figure S7. Time dependent ^1H NMR spectra of 500µM **3** in PBS with 47% D_2O and 20% CD_3CN recorded over 24 h at room temperature. Signals are referred to the signal of CD_2HCN set at δ= 2.13 ppm.

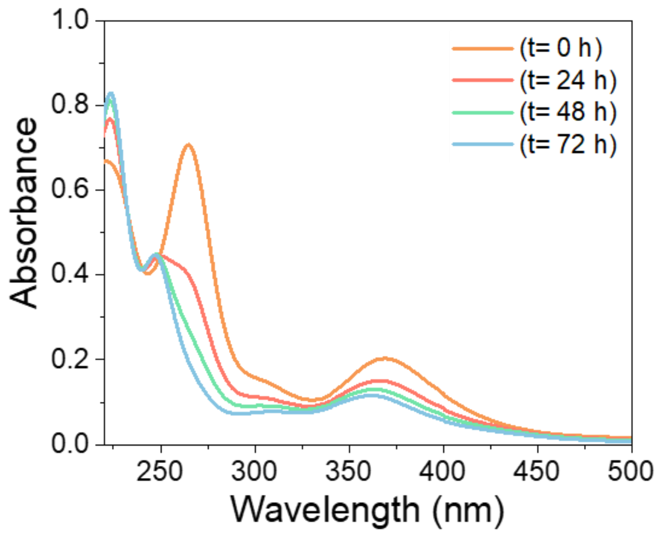

Figure S8. Time dependent UV-Vis spectra of 30μM **4** in PB (50mM) with 0.6 % CH_3CN.

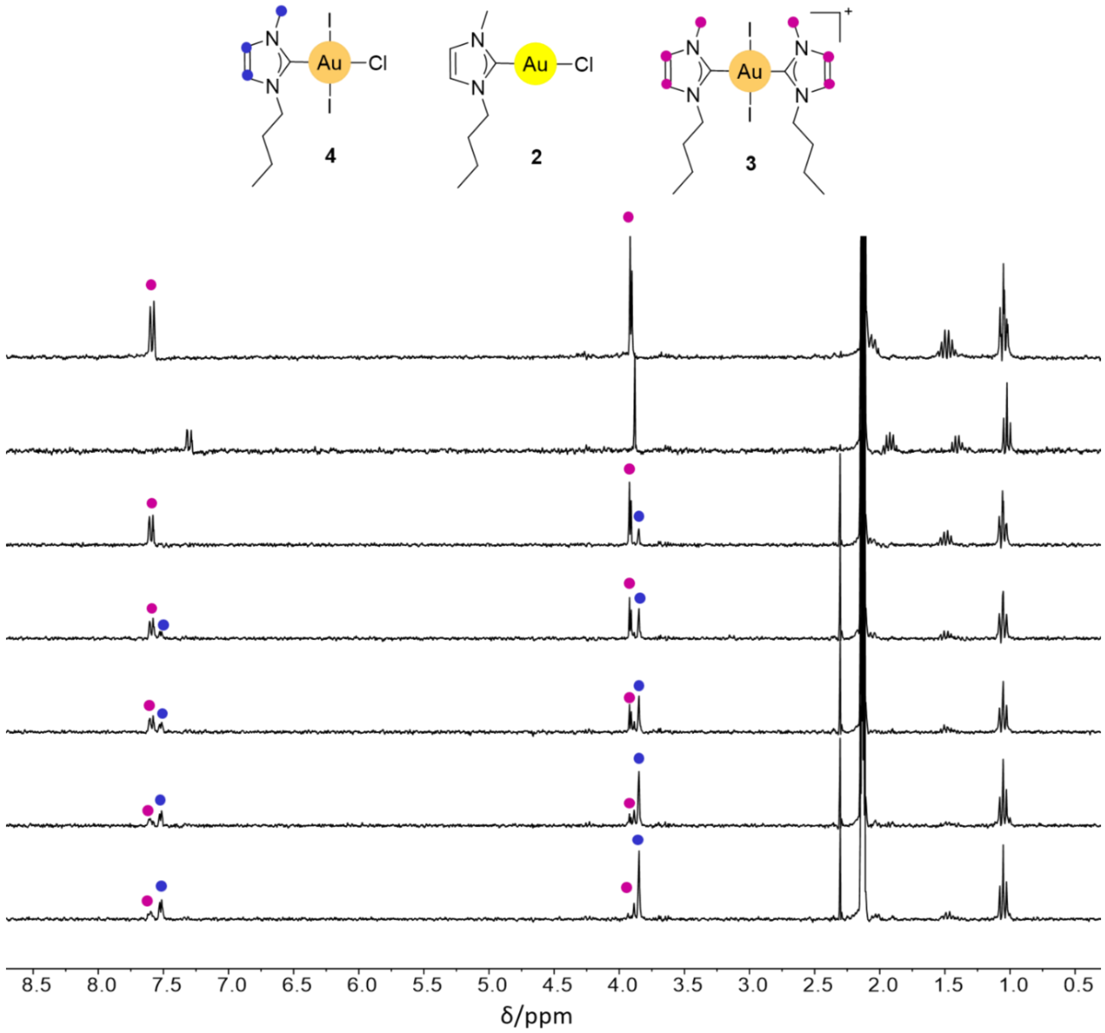

Figure S9. Watergate ^{1}H NMR spectra of 500µM **4** in PBS with 47% D_2O and 20% CD_3CN recorded over 24 h, compared with the corresponding spectra of the reduced form **2** and the biscarbenic compound **3**. Signals are referred to the signal of CD_2HCN set at δ= 2.13 ppm.

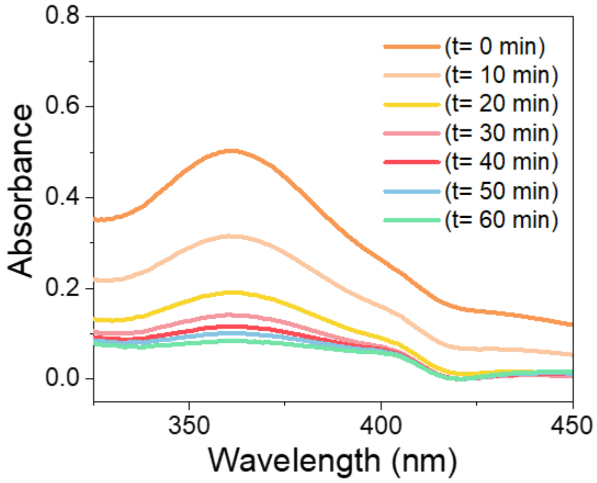

Figure S10. UV-Vis spectra of 150µM **3** in PBS + 10% FBS and 5 % DMSO, recorded over 1 h.

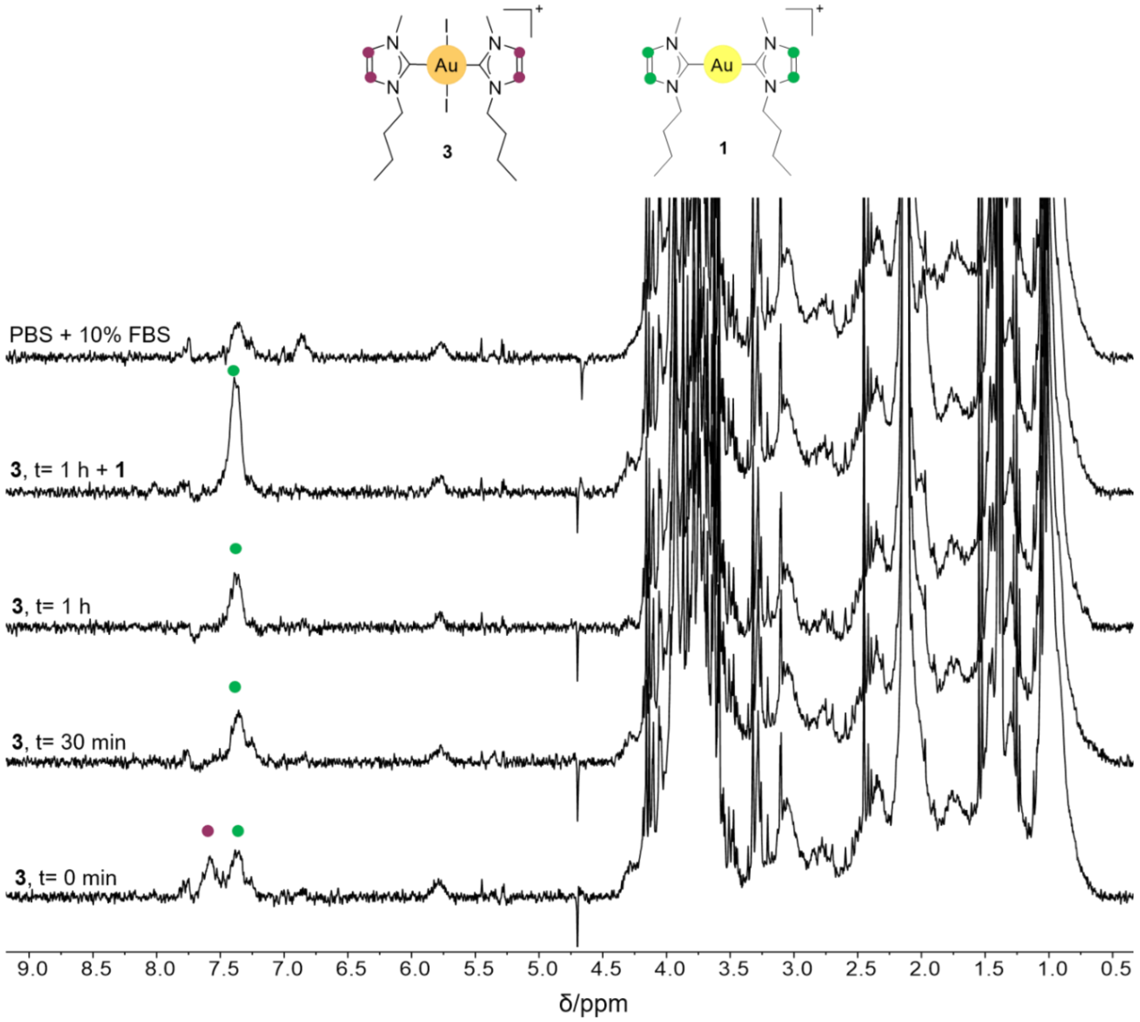

Figure S11. Watergate ^{1}H NMR spectra of 333μM **3** in PBS + 10% FBS with 37% D_2O and 20% CD_3CN, recorded over 1 h. After 1 h, the solution is spiked with **1** (final concentration 333μM). Signals are referred to the residual signal of CD_2HCN set at δ= 2.13 ppm.

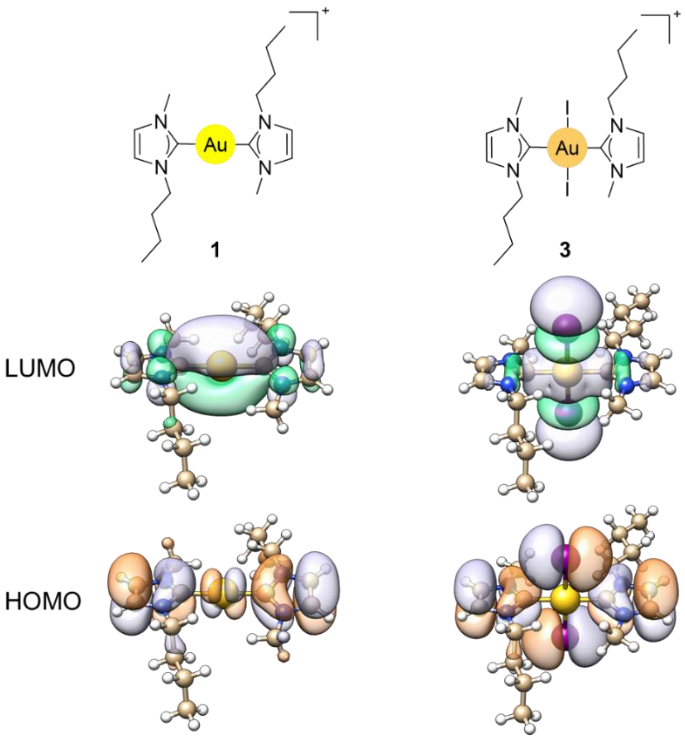

Figure S12. DFT calculated HOMO and LUMO orbitals for compounds **1** and **3** in their *anti* conformations. DFT calculations determined an energy difference between the *syn* and *anti* isomers of only 0.3 kJ/mol, with the *syn* structure more stable than the *anti*.

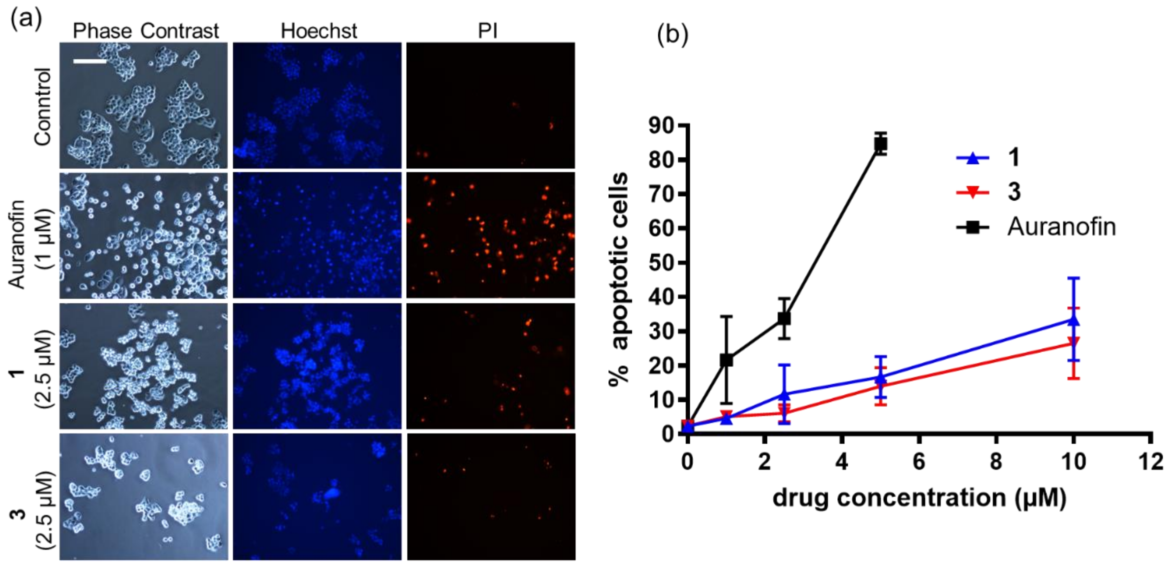

Figure S13. (a) Fluorescent microscopy images of Hoechst/PI double staining experiments. A2780 cells were left untreated (control) or treated with auranofin, **1** or **3** at the indicated

concentrations for 24 h. Hoechst (blue) stains DNA and PI (red) dead cells. Early apoptosis is characterized by condensed chromatin (intense blue staining) without PI accumulation and late apoptosis by condensed chromatin with PI staining. Scale bars: 200 µm (b) Auranofin induced higher levels of apoptosis as compared to the Au-NHC compounds. Apoptotic cells were detected by Hoechst-PI double staining as published.[4]

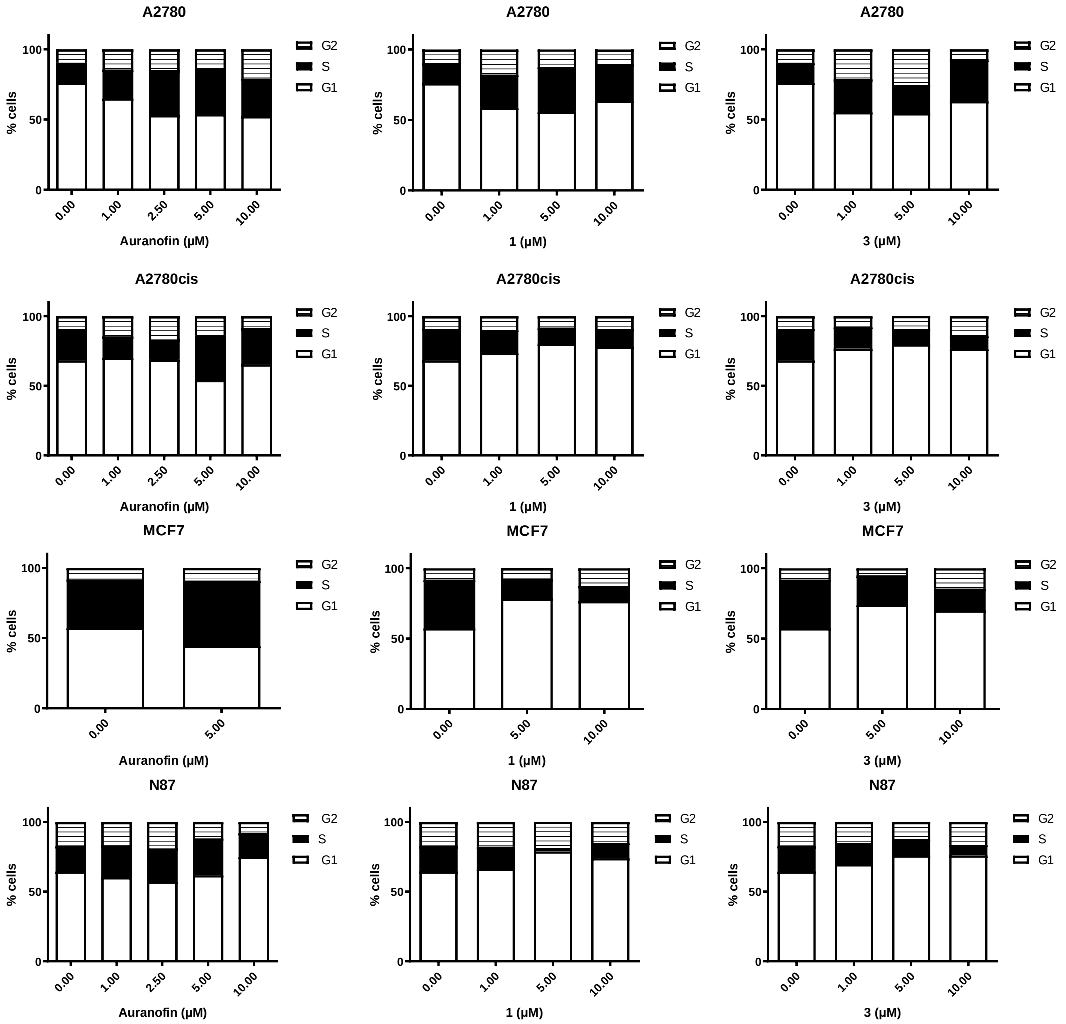

Figure S14. Impact of the investigated Au compounds on cell cycle distribution. The indicated cell models were exposed to increasing concentrations of auranofin (left), **1** (middle), and **3** (right), for 24 h and cell cycle distribution was analysed by FACS sorting of PI-stained cells. One representative experiment is shown.

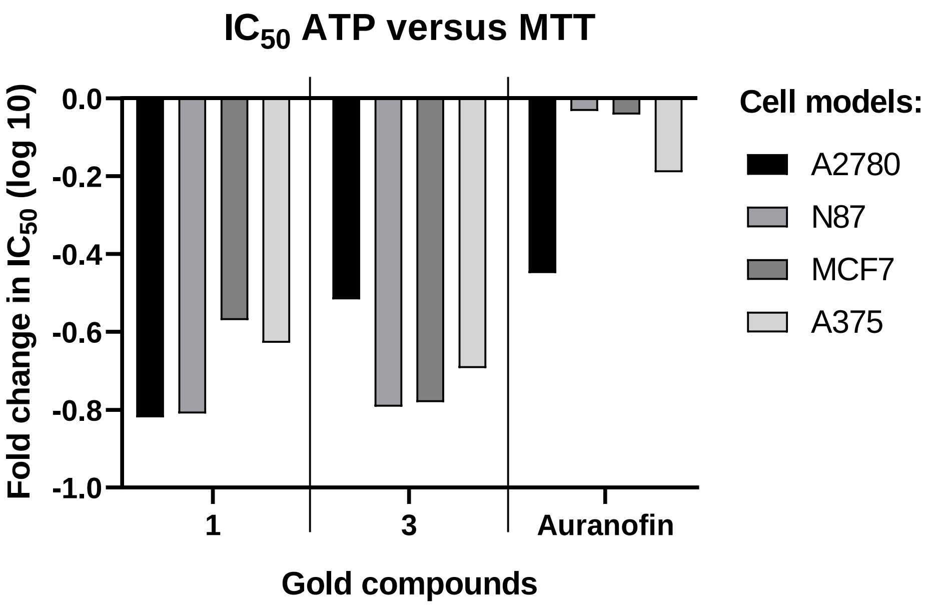

Figure S15. Change in IC_{50} values determined by an MTT based viability assay as compared to ATP content measured by CellTiter-Glo assay. Mean IC_{50} values for the ATP assay were divided by the ones for MTT assay (compare also Table 1) and the values are given as log10.

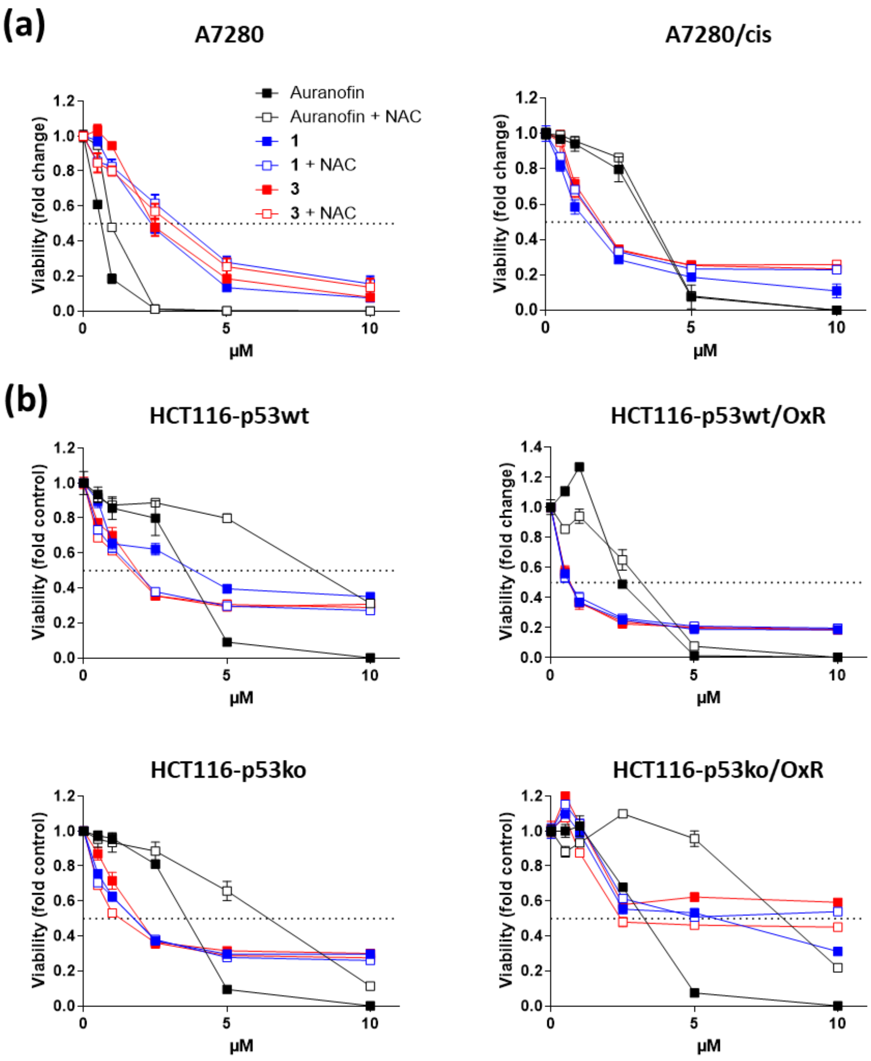

Figure S16. Impact of ROS scavenging by NAC (1 mM) on the anticancer activity of the investigated gold compounds as indicated in the (A) ovarian cancer cell lines A2780 and its cisplatin-resistant subline and the (B) colorectal carcinoma cell line HCT116. In this model two sublines with differing p53 status (wild-type = wt; deleted = ko) and the respective oxaliplatin-resistant subclones (OxR) are compared.

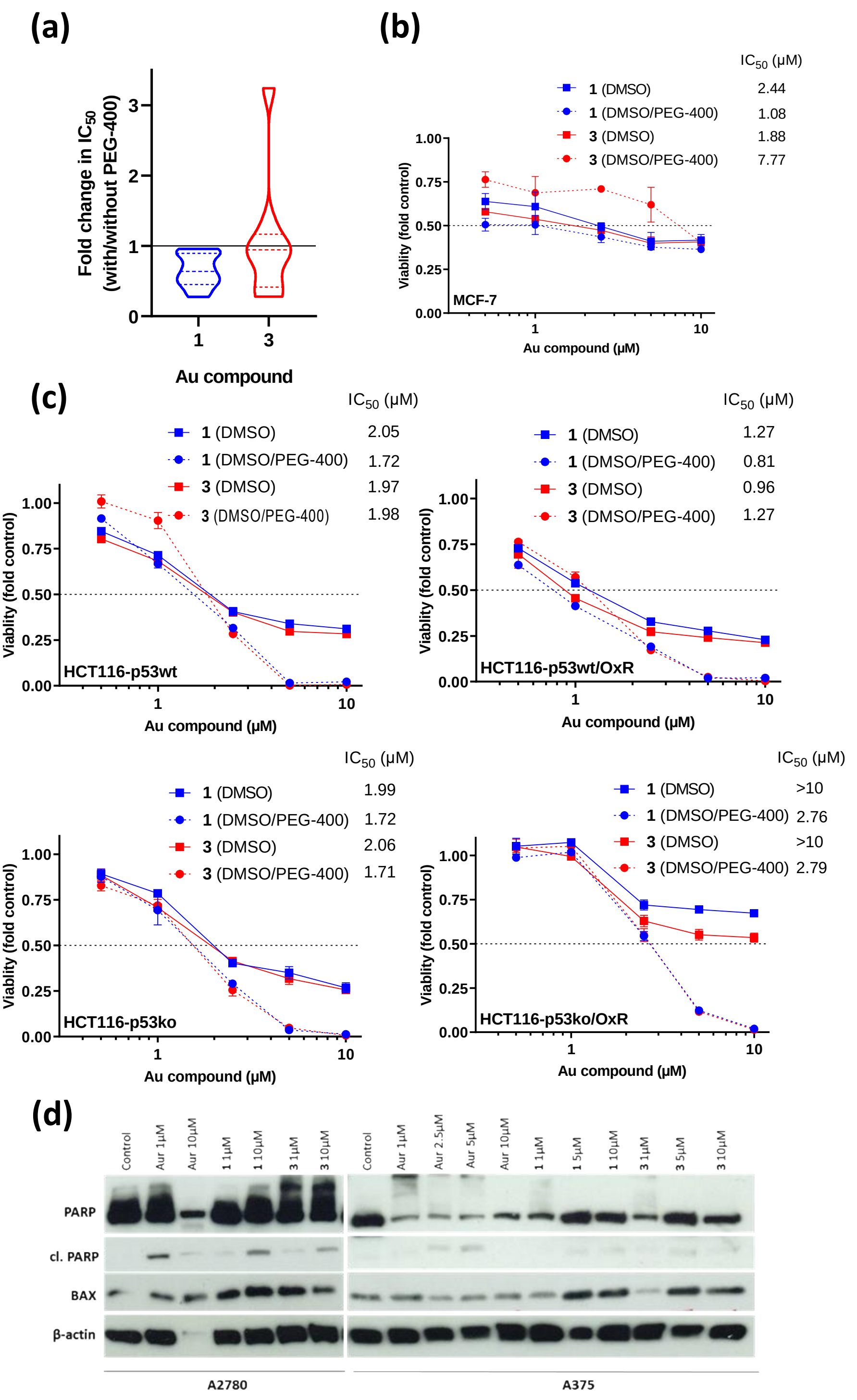

Figure S17. Impact of PEG-400 formulation on the anticancer activity of **1** and **3** as analysed by cell viability assays. (a) Fold change in IC_{50} values for **1** (blue) or **3** (red) in DMSO/PEG-400-based *vs.* standard DMSO-based formulation are depicted as violin blots. Values for all cell models shown in Table 1 derived from at least two independent experiments in triplicate were included. (b,c) Representative examples for dose-response curves of **1** (blue) or **3** (red) formulated as indicated for MCF-7 (b) and the entire HCT116 cell model family (c). (d) Impact of auranofin *vs.* **1** and **3** on expression/cleavage of the apoptosis indicator PARP1 and the pro-apototic bcl2 protein family member bax in the indicated cell models. β-actin (blotted at a separate membrane due to size interference) was used as loading control. Note general protein degradation at 10 µm auranofin due to massive cell death induction in A2780 cells.

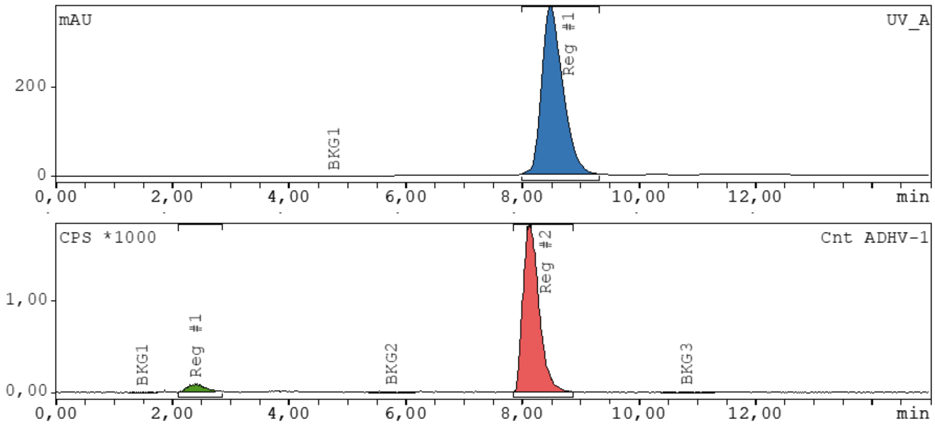

Figure S18. HPLC chromatogram of [^{124}I]**3** after purification**.** Both signals obtained from UV detector (top) and radioactivity detector (bottom) are shown. Radiochemical purity was calculated from the chromatographic profile, as the ratio between the area under the peak corresponding to [^{124}I]**3** (in red) and the sum of the areas under all peaks in the chromatogram.

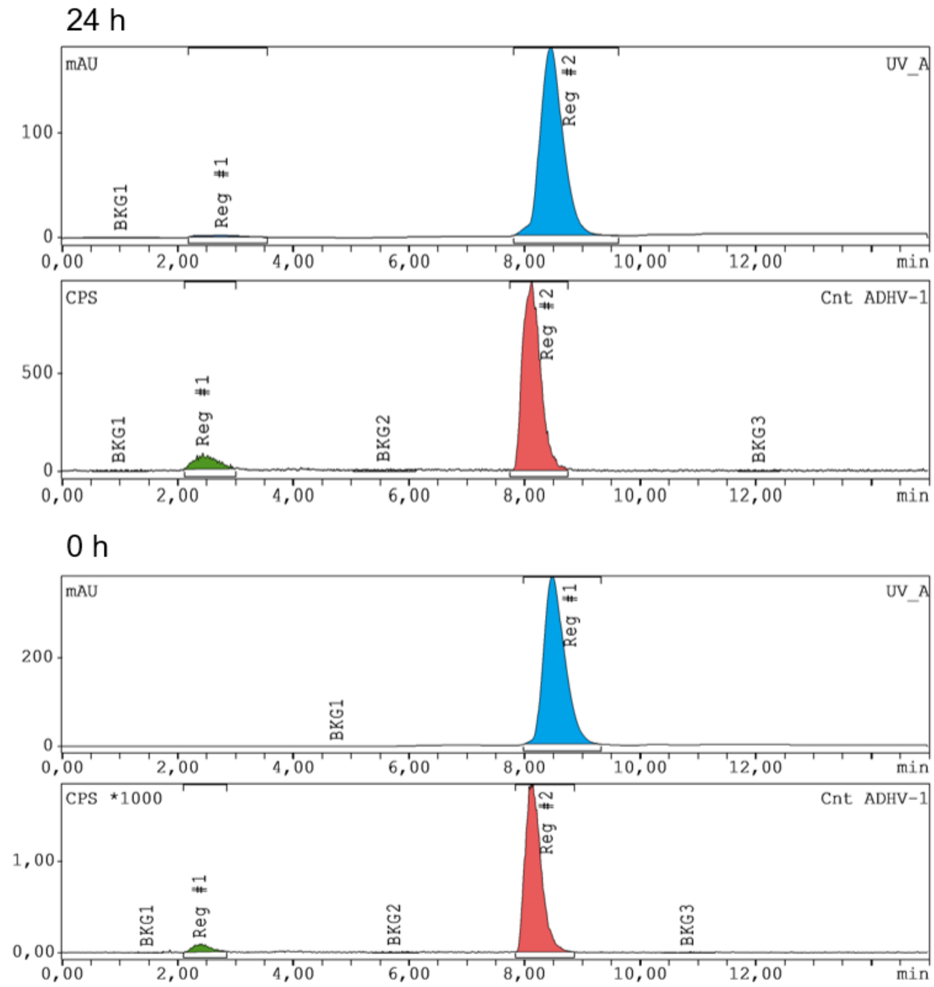

Figure S19. HPLC chromatogram of [^{124}I]**3** at t = 0 and t = 24 h after incubation in the formulation. Both signals obtained from UV detector (top) and radioactivity detector (bottom) are shown.

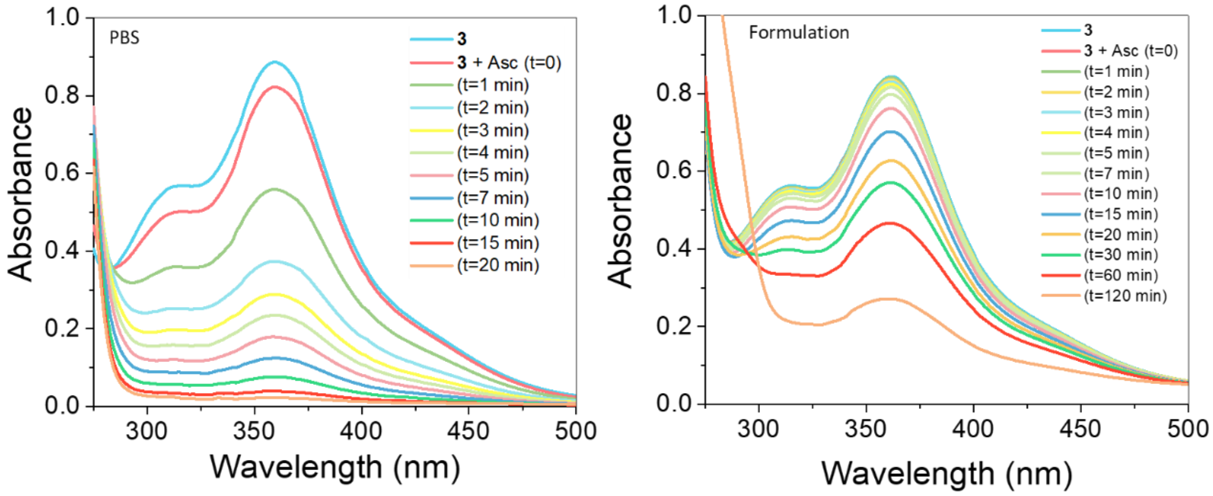

Figure S20. Time dependent UV-Vis spectra of 150µM **3** in PBS (left panel) and PEG-based formulation (PBS with 30% PEG and 5% DMSO) (right panel) in the presence of equimolar amount ascorbic acid under light constant stirring conditions.

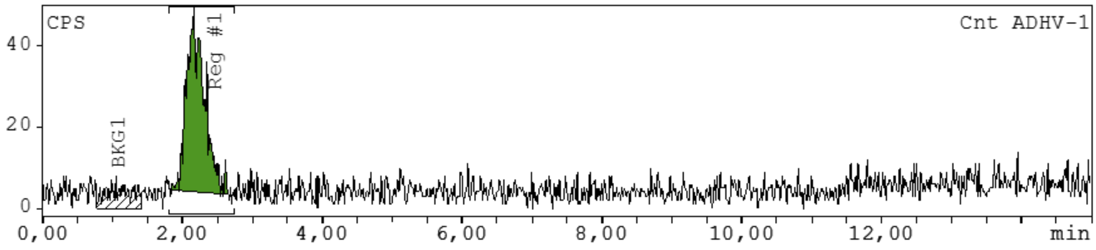

Figure S21. HPLC chromatogram (radioactive detector) obtained from a blood sample extracted at t = 4 min after intravenous administration of [^{131}I]**3**.

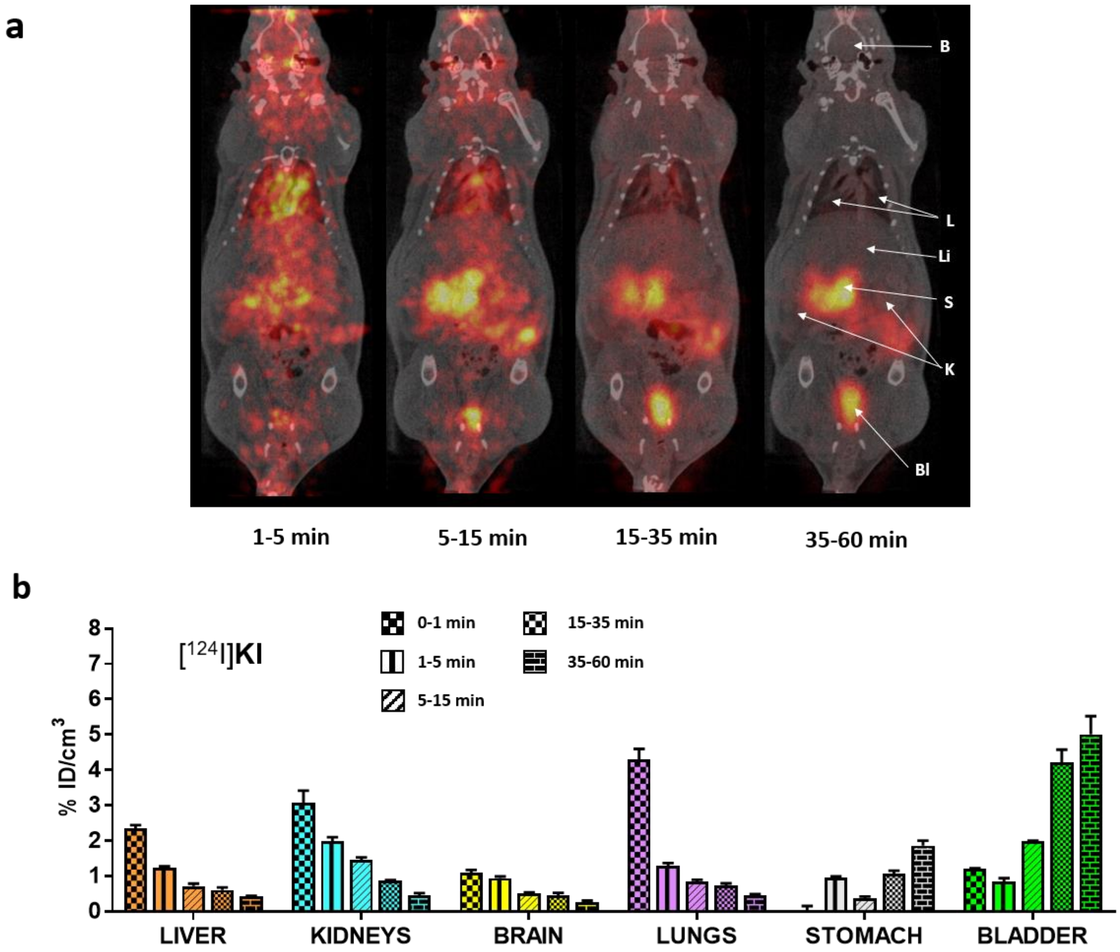

Figure S22. (a) PET images (maximum intensity projections) obtained at different time intervals after intravenous administration of [^{124}I]KI. PET images have been co-registered with representative CT slices for localization of the radioactive signal. (b) Concentration of radioactivity in different organs and at different times after intravenous administration of [^{124}I]KI, as determined from PET images. Histograms are obtained by averaging the %ID/g values for the indicated time frame.

Experimental procedures

Synthesis and characterization of Au(III) NHCs

General aspects

If not stated otherwise solvents and reagents were used in commercial grade without purification. Potassium iodide was purchased from Fisher Scientific and used as received. Other chemicals were purchased from Sigma Aldrich, deuterated solvents from Cortecnet

while other HPLC grade solvents were purchased from Scharlab (Sentmenat, Barcelona, Spain). Au(I) carbenes **1** and **2** were synthesized according to procedures already reported by some of us.[5]

^{1}H and ^{13}C NMR spectra were recorded on a Bruker Fourier TM 300 instrument equipped with Sample Xpress system or on a Bruker 500 Avance equipped with a z gradient BBOF probe. Spectra were analysed using Mestrenova 12 (MestreLab Research S.L). Mass spectrometric analysis of the compounds was carried out on an Aquity Ultra Performance Liquid Chromatography (UPLC) separation module, coupled with a LCT-TOF Premier XE mass spectrometer (Waters, Manchester, UK). Reverse phase C18 column (50×2.1 mm, 1.7 µm particle size, Waters) was used as the stationary phase. Elemental analyses have been performed on a LECO® microanalyzer.

Synthesis of bis(1-butyl-3-methyl-imidazol-2-ylidene)-gold-diiodide hexafluorophosphate (3)

Compound **3** was obtained by adapting a procedure reported by Baron et al. for dinuclear Au(III) NHC complexes.[6]

The gold(I) precursor **1** (50 mg, 0.08 mmol) was dissolved in 7 ml of acetonitrile. 2.5 equivalents of I_2 (51 mg, 0.2 mmol) were added from a freshly prepared 0.5 M stock solution in acetonitrile. The reaction was protected by light. After 1 h solvent was removed under reduced pressure, the resulting orange solid was washed with hexane to remove the excess of I_2 and dried under vacuum. (60 mg, 86% yield). Single crystals suitable for x-ray diffraction analysis were obtained by stratification of diethyl ether onto a concentrated solution of **3** in acetonitrile.

^{1}H NMR (300 MHz, CD_3CN): δ (ppm)= 7.41 (m, 2H, NCHCHN); 7.39 (m, 2H, NCHCHN); 4.16-4.11 (m, 4H, N$\mathit{CH_2}$$CH_2CH_2CH_3$); 3.78 (s, 3H, NCH_3); 3.77 (s, 3H, NCH_3); 1.99-1.91 (m, 4H, $NCH_2$$\mathit{CH_2}$$CH_2CH_3$); 1.45-1.34 (m, 4H, $NCH_2CH_2$$\mathit{CH_2}$$CH_3$); 0.96 (t, J= 7.44 Hz, 6H, $NCH_2CH_2CH_2$$\mathit{CH_3}$). ^{13}C NMR (125 MHz, CD_3CN): δ (ppm)= 144.6 (NCN); 144.6 (NCN); 126.8 (NCHCHN); 126.8 (NCHCHN); 125.3 (NCHCHN); 125.3 (NCHCHN); 51.7 (N$\mathit{CH_2}$$CH_2CH_2CH_3$); 51.7 (N$\mathit{CH_2}$$CH_2CH_2CH_3$); 38.8 (N$\mathit{CH_3}$); 38.7 (N$\mathit{CH_3}$); 32.5 ($NCH_2$$\mathit{CH_2}$$CH_2CH_3$); 32.4 ($NCH_2$$\mathit{CH_2}$$CH_2CH_3$); 20.4 ($NCH_2CH_2$$\mathit{CH_2}$$CH_3$); 20.3 ($NCH_2CH_2$$\mathit{CH_2}$$CH_3$); 13.8 ($NCH_2$$\mathit{CH_2}$$CH_2$$\mathit{CH_3}$). ESI-TOF-MS ($CH_3CN$-$H_2O$): *exact mass* for $[C_{16}H_{28}N_4I_2Au]^+$ 727.0069: measured *m/z* 726.9992. Anal. Calc. for $C_{16}H_{28}N_4I_2AuPF_6$: C: 22.12, H: 3.11, N: 6.39; Found: C: 22.03, H: 3.24, N: 6.42.

Synthesis of 1-butyl-3-methyl-imidazol-2-ylidene-gold-chloride-diiodide (4)

The synthetic procedure was the same followed for **3**. Complex **2** (50 mg, 0.135 mmol), I_2 (85 mg, 0.338 mmol). After 1 h stirring at room temperature, the reaction mixture was filtered on celite to remove what seemed to be Au(0) (black powder). Solvent removal under reduced pressure gave an oily solid residue. The residue was dissolved in a little amount of DCM and precipitated with hexane to obtain a purple solid. (41 mg, 48% yield). Single crystals suitable for x-ray diffraction analysis were obtained by stratification of hexane onto a concentrated solution of **4** in dichloromethane.

^{1}H NMR (300 MHz, CD_3CN): δ (ppm)= 7.39 (d J^3 = 2.09 Hz, 1H, NCHCHN); 7.37 (d J^3 = 2.09 Hz, 1H, NCHCHN); 4.10 (t ^{3}J = 7.44 Hz N$\mathit{CH_2}$$CH_2CH_2CH_3$); 3.72 (s, 3H, NCH_3); 1.97-1.87 (m, 2H, N$\mathit{CH_2}$$CH_2$$\mathit{CH_2}$$CH_3$); (m, 2H, $NCH_2CH_2$$\mathit{CH_2}$$CH_3$); 1.40 (sextet ^{3}J = 7.44 Hz, 2H, $NCH_2CH_2$$\mathit{CH_2}$$CH_3$); 0.96 (t ^{3}J = $NCH_2CH_2CH_2$$\mathit{CH_3}$. ^{13}C NMR (125 MHz, CD_3CN): δ (ppm)= 127.0 (NCHCHN); 125.4 (NCHCHN); 51.4 (N$\mathit{CH_2}$$CH_2CH_2CH_3$) 39.1 (N$\mathit{CH_3}$); 31.8 ($NCH_2$$\mathit{CH_2}$$CH_2CH_3$); 20.2 ($NCH_2CH_2$$\mathit{CH_2}$$CH_3$); 13.8 ($NCH_2$$\mathit{CH_2}$$CH_2$$\mathit{CH_3}$).

Anal. Calc. for $C_8H_{14}AuN_2ClI_2$: C: 15.39, H: 2.26, N: 4.49; Found: C: 15.52; H: 2.17; N: 4.69.

X-ray crystallography

Data collections of the compounds reported were performed at the X-ray diffraction beamline (XRD1) of the Elettra Synchrotron (Trieste, Italy), with a Pilatus 2M image plate detector. Complete datasets were collected at 100 K (nitrogen stream supplied through an Oxford Cryostream 700) with a monochromatic wavelength of 0.700 Å with the rotating crystal method. The diffraction data were indexed, integrated and scaled using XDS.[7] The structures were solved by direct methods using SIR2014.[8] Fourier analysis and refinement were performed by the full-matrix least-squares methods based on F^2 implemented in SHELXL-2014.[9] The Coot program was used for modeling.[10] Anisotropic thermal motion was allowed for all non-hydrogen atoms. Hydrogen atoms were included at calculated positions with isotropic factors U = 1.2 Ueq, Ueq being the equivalent isotropic thermal factor of the bonded non hydrogen atom. Crystallographic and refinement data are reported in Table S1.

Stability of the Au(III) complexes in solution

Milli-Q (MQ) water was purified with a Millipore Direct-Q® 3 UV apparatus. Buffers were prepared by dissolving the suitable salts in MQ water and adjusting the pH. Concentrated stock solutions of the compounds (5 mM or 10 mM) were freshly prepared in DMSO, in deuterated or non-deuterated acetonitrile and then diluted in the suitable buffers. UV-Vis absorption spectra were recorded on a Jasco V-730 spectrophotometer. All spectra were recorded using quartz cuvettes (Hellma Analytics) of 1000 µL or 2000 µL volume and an optical path of 1 cm.

Stability of **3** and **4** in phosphate buffer (50 mM, pH= 7.4) was first investigated by UV-Vis absorption spectroscopy: spectra were carried out protecting the sample from light and monitoring spectral changes at t= 0 min, 90 min, 24 h, 48 h, 72 h.

Stability of the complexes in PBS was evaluated through ^{1}H NMR spectroscopy: water suppressed spectra of **3** (250 µM) and **4** (1 mM) in PBS with 47% D_2O and 20% CD_3CN were recorded at t= 0, 1, 3, 6, 24 h with water suppression (Watergate) settings and 64 scans. The signal of CD_2HCN was set as reference to compare spectra. Reactivity of the compounds in PBS supplemented with 10% FBS was monitored by means of UV-Vis absorption spectroscopy. FBS was purchased from Fisher Scientific. Besides, proton NMR experiments of **3** (333 µM) in PBS + 10% FBS with 37% D_2O 20% CD_3CN were carried out (Watergate, 64 scans). The residual signal of CD_2HCN was set at δ= 2.13 as the reference to compare spectra. All Data were analyzed and plotted using OriginPro 2016 (Origin Lab Corp) and Mestrenova 12 (MestreLab Research S.L).

DFT calculations

Calculations were performed using Gaussian16 package.[11] Geometry optimization was carried out using pbe1 algorithm[12] and CEP-31G functional basis sets.[13] Solvent was considered by means of the conductor like polarized continuum model (CPCM) with water as the implicit solvent.[14] Geometry was optimized and frequency calculations were carried out to avoid the presence of imaginary modes. Results have been compared with RX structures to check the suitability of the model.

In vitro experiments

Cell culture

The human cancer cell lines MCF-7, A375, and N87 were purchased from American Type Culture Collection (Manassas, VA, USA). A2780 and A2780cis were purchased from Sigma-Aldrich (MO, USA). HCT116-p53wt and HCT116-p53ko were kindly donated by Dr. B. Vogelstein (John Hopkins University, Baltimore, USA, and the respective oxaliplatin-resistant HCT116 sublines were generated in the lab of Walter Berger at the Institute of Cancer Research (Vienna, Austria).[15] All cell lines were cultured in growth media (MCF-7 in DMEM; A375, N87, and A2780 in RPMI 1640; HCT116 in McCoy`s medium) supplemented with 10% fetal bovine serum (FBS South America, Biowest, Nuaillé, France) at 37 °C and 5% CO_2. Cells

were regularly screened for *Mycoplasma* contamination (Mycoplasma Stain kit, Sigma, St. Louis, Missouri, USA).

Cell viability assays (MTT and ATP assays)

These assays were performed as published.[16] Shortly, 2-4x10^4 cells/ml were seeded into 96-well plates, left to adhere overnight, then treated with the indicated concentrations of auranofin, **1**, **3,** or oxaliplatin for 72 h. Gold drugs were standardly dissolved in DMSO (10mM stock solutions for **1**, **3**, and auranofin). Oxaliplatin was dissolved in H_2O (10mM stock solution). For analysis of the activity of **1** and **3** dissolved in the PEG-400-based formulation, stock solutions were prepared using a mixture of 30% PEG-400, 5% DMSO, and 65% PB instead of DMSO. For testing the effect of N-acetylcysteine (NAC) and ascorbic acid (AA) on the anticancer activity of the gold compounds**,** cells were pre-incubated with 1 mM NAC or 50 µM AA for 1h before the respective gold drugs were added for 72 h. For serum reduction experiments, cells treated with the indicated gold drugs were incubated in growth medium containing 0.1% instead of 10% FBS. For analysis of the influence of hypoxic conditions, cells treated with the indicated gold drugs were incubated under 1% oxygen in a Heracell 150i incubator (Thermo Scientific). Cell viability was either determined using the 3-(4,5-dimethylthiazol-2-yl)-2,5-diphenyltetrazolium bromide (MTT)-based vitality assay (EZ4U, Biomedica, Vienna, Austria), measuring mitochondrial activity by reduction of tetrazolium salts into formazan derivatives, or by CellTiter-Glo® Luminescent Cell Viability Assay (Promega), measuring mitochondrial activity by detection of cellular ATP contents, in both cases following the instructions of the manufacturer. IC_{50} values (corresponding to drug concentrations leading to a viability reduction by 50% as compared to the untreated control) were calculated from whole dose-response curves generated by GraphPad Prism (version 8.0.1) software. IC_{50} values given are derived from at least three independent experiments in triplicate. In the representative dose-response curves shown, each data point represents the mean ± SD of the respective triplicate.

Hoechst 33258/propidium iodide (HoePI) staining.

Hoechst 33342/propidium iodide (Hoe/PI) double staining for detection of (early as well as late) apoptotic cells was performed as described in detail by Grusch et al.[4] A2780 cells were seeded in duplicates (1.25x10^4 cells per well) into 24 well plates, allowed to recover overnight, and treated with the indicated concentrations of gold compounds for 24h. Hoechst (1 µg/ml) / PI (2.5 µg/ml) was added for 1 h and pictures were taken on a Nikon eclipse Ti-e fluorescence microscope with a sCMOS pco.edge camera (100x magnification). Data evaluation was

performed by counting the number of all cells as well as early and late apoptotic cells using the ImageJ software.

Cell cycle analysis by flow cytometry

3-5x10^5 cells per well were seeded into 6 well plates, allowed to recover for 24h, and treated with the indicated concentrations of auranofin, **1**, or **3** for 24 h. Afterwards, propidiumiodide-based DNA content analysis (PI-staining) was performed as published.[17] PI fluorescence intensity measurements were carried out on a LSRFortessa flow cytometer (BD Biosciences, East Rutherford, NJ,USA) and data were analysed by Flowing Software 2.5.1 (Perttu Terho, Turku, Finland).

Clone formation assay

5x10^4 A2780 cells were incubated for 2h in PBS supplemented with calcium and magnesium (HyClone, GE Healthcare) with the indicated concentrations of **1** or **3** with/without 50µM ascorbic acid. Then, cells were centrifuged, the supernantant was removed, and the cell pellet was resuspended in 700 µL growth medium containing 10% FBS. Either 100 µL or 200 cells/well (7x10^3 or 14x10^3 cells/well) were seeded in duplicates into 24 well plates and further incubated to test for clone formation ability. Cell clones were visualized after 10 days by crystal violet staining as described[16] and scanned on a Typhoon scanner (Typhoon TRIO Variable Mode Imager, GE Healthcare Life Sciences). Afterwards, crystal violet was dissolved again by 2% SDS (in H_2O), and absorbance (corresponding to the number of adherent cell clones) was measured at 560 nm on a Tecan infinite M200 pro spectrophotometer.

Western blot analysis

Western blot experiments were performed as published. In short, the indicated cell models were exposed in 6-well plates to increasing concentrations of the indicated gold compounds for 24 h before prepartation of total protein extracts for immunoblotting.[17]

Radiochemical synthesis of compound 3

General aspects

Na[^{131}I]I (70 MBq, solution in 0.1 M NaOH) and Na[^{124}I]I (370 MBq, solution in 0.02 M NaOH) were obtained from Perkin Elmer. HPLC was carried out using an Agilent 1200 series system, equipped with a variable wavelength UV detector and a radioactive detector (Gabi, Raytest) connected in series. A Mediterranea SEA 18 column (5 µm, 150 × 0.46) was used as the

stationary phase. The amount of radioactivity in samples to determine the radiochemical yield was measured with a dose calibrator (Carpintec CRC-25, USA). The radiochemical yield was calculated as the percentage of activity in the final product compared to the initial amount of [^{124}I]KI reacted.

Protocol for the preparation of radiolabeled 3

A stock of KI 0.28 M in HCl 2.9 M was prepared. 11.5 µL of this stock (KI 3.23 µmol, 5 eq) were spiked with 5 µL of [^{124}I]I^- (315 µCi, 11.6 MBq) in NaOH 0.1 M. The solution was reacted with 1 µL of H_2O_2 (30% v/v, 9.8 µmol, 15 eq). After 45 min, the obtained solid iodine was washed with 50 µL of H_2O for three times. 133.4 µL of a 4.85 mM stock solution of **1** in acetonitrile (0.65 µmol, 1 eq) were reacted with the iodine solid. After 2 h the solvent was removed, and the product was washed twice with 100 µL of hexane. For analysing the purity, the solid was dissolved in 100 µL of CH_3CN (HPLC grade). 10 µL of this solution were taken and diluted in 20 µL of a 1:1 mixture of CH_3CN/H_2O. 20 µL of the obtained solution were then injected in the HPLC instrument. Compound **3** was obtained within a 95% radiochemical purity and a 35% radiochemical yield.

In vivo experiments

General aspects

Animals were maintained and handled in accordance with the Guidelines for Accommodation and Care of Animals (European Convention for the Protection of Vertebrate Animals Used for Experimental and Other Scientific Purposes). All animal procedures were performed in accordance with the Spanish policy for animal protection (RD53/2013), which meets the requirements of the European Union Animal Directive (2010/63/EU). Experimental procedures were approved by: (i) Ethical Committee of CIC biomaGUNE (project number AE-biomaGUNE-0216); (ii) Órgano Habilitado del IIS Biodonostia; and (iii) local authorities (Diputación Foral de Guipuzcoa; project code PRO-AE-SS-059). Experimental procedures were carried out by accredited personnel (Jordi Llop, Vanessa Gómez-Vallejo, Rossana Passannante) in the AAALAC accredited Animal Facility of CIC biomaGUNE (Authorisation code: ES200690050402).

For in vivo studies, compound **3** was dissolved in 1000 µL mixture of 5% DMSO, 30% PEG400 in PBS to a final concentration of 640 µM. Stability in this formulation over 24 h was evaluated through UV-Vis absorption spectroscopy and HPLC analysis. For UV-Vis experiments solutions of 150 µM **3** where prepared in the formulation alone or with addition of 10% FBS, protected from light and analysed over 24 h or 1 h respectively. For HPLC analysis, 10 µL of

^{124}I-radiolabelled **3** in formulation were taken and diluted in 20 µL of a 1:1 mixture of CH_3CN/H_2O. 20 µL of the obtained solution were injected into the HPLC. For the analysis, an Agilent 1200 series HPLC system equipped with a UV-Vis and a radioactivity detector (Gabi, Raytest) was used. A Mediterranea Sea18 column (4.6x150 mm, 5 µm particle size, Teknokroma, Spain) was used as the stationary phase and MQ water with 1% trifluoroacetic acid (TFA); B: acetonitrile; flow rate = 1mL/min were used as the mobile phase.

Pharmacokinetic Study

Anesthesia was induced to rats with 5% isoflurane and maintained by 2% of isoflurane in 100% O_2. To perform the surgery, rats were positioned in supine position and the two distal hind limbs were fixed forming an approximately 30° angle with the horizontal plane. A skin incision was made in the femoral region. Soft tissue was dissected to expose the femoral neurovascular bundle, and fine tip forceps were placed between the artery, vein and nerve and slowly opened to separate the vessels. The vena profunda femoris was sutured to avoid retrograde bleeding via this vessel, and the sapheno-femoral junction was cauterized. A few millimeters from venatomy, a vascular clip was inserted in the direction of the catheter (to control the flow) and a suture was applied in the opposite direction (to avoid retrograde bleeding). The same operation was carried out both on the femoral vein and femoral artery. An appropriate syringe filled with 50 U/ml Heparin/physiologic saline solution was fixed at the terminus of the catheters and these were filled with heparinized saline solution. On the upper half of the vessel circumference, at a 45° angle, an incision was done and the catheter (fine bore polythene tubing: ID 0.58 mm, OD 0.98 mm) was inserted through the venotomy and advanced to the vascular clip. The vascular clip was then removed and the catheter advanced distally towards the level of the inguinal ligament. The catheter was finally secured to the femoral vein/artery proximally and distally with 2 single knots using 4-0 braided silk suture.

The catheters were connected to an in-house developed system composed by a peristaltic pump and gamma-radiation coincidence detector (Bioscan, B-FC-4100). The femoral artery catheter, the gamma detector, the peristaltic pump and the femoral vein catheter were thus connected in series, to enable the extracorporeal circulation of blood (flow 150 µL/s from femoral artery to femoral vein) and continuous measurement of radioactivity in the blood. Data was imported in real time to an excel sheet using PLX-DAX add-on for Microsoft Excel (measured in volts per second).

The labeled compound (ca. 1.11 MBq, 300 µL, 640 µM) was injected concomitantly with the start of the acquisition through the femoral vein.

At different time points arterial blood samples (150 µL) were withdrawn. Blood samples were processed to separate the plasma, which was further processed to determine the presence of radioactive metabolites. With that aim, plasma fractions were diluted with the same volume of

acetonitrile. After mixing vigorously for 20 s, samples were centrifuged at 14800 rpm for 4 minutes. The liquid phase was separated from the precipitate by decantation and was injected into the HPLC system, using the same conditions as for quality control.

In vivo PET Studies

PET studies were carried out in rats (n=2 per compound) using an eXplore Vista-CT small animal PET-CT system (GE Healthcare). Anaesthesia was induced with 5% isoflurane and maintained by 1.5 to 2% of isoflurane in 100% O_2. For intravenous administration of the radiotracer, the tail vein was catheterized with a 24-gauge catheter and the labelled compounds (1.2 MBq for [^{124}I]**3**; 1.9 MBq for [^{124}]KI; volume=300 µl) were injected concomitantly with the start of a PET dynamic acquisition. Mice were kept normothermic throughout the scans using a heating blanket (Homeothermic Blanket Control Unit; Bruker).
All the scans were recorded in the 400-700 KeV energetic window. CT acquisitions were also performed at the end of each PET scan, providing anatomical information for unambiguous localization of the radioactive signal. After the imaging session, animals were sacrificed and organs were harvested for further processing (only for the case of [^{124}I]**3**).
PET images were reconstructed (decay and CT-based attenuation corrected) with filtered back projection (FBP) using a Ramp filter with a cut off frequency of 1 Hz. Images were analyzed using PMOD image analysis software (PMOD Technologies Ltd, Zürich, Switzerland). With that aim, frames acquired in the tame ranges corresponding to 0-1 min, 1-5 min, 5-15 min, 15-35 min and 35-60 min were averaged and volumes of interest (VOIs) were manually drawn in different organs (liver, kidneys, bladder, brain, lungs and stomach) using the CT images as anatomical reference. VOIs were then transferred to the PET images and time activity curves (decay corrected) were obtained for each organ as cps/cm^3. Curves were transformed into real activity (Bq/cm^3) curves. Injected dose normalization was finally applied to data to get time activity curves as percentage of injected dose per cm^3 of tissue.

ICP Analysis.

After complete decay of the radioactivity, the extracted organs were digested in aqua regia overnight at 70°C. The samples were filtered through cotton and diluted with 2% HNO_3 to enable ICP-MS analysis. ICP-MS measurements were performed on a Thermo iCAP Q ICP-MS (Thermo Fisher ScientificGmbH, Bremen, Germany). An ASX-560 autosampler was coupled to the ICP-MS (CETAC Tech, Omaha, NE, USA).

Bibliography

[1] R. Jothibasu, H. V. Huynh, L. L. Koh, *J. Organomet. Chem.* **2008**, *693*, 374–380.
[2] A. K. Ghosh, V. J. Catalano, *Eur. J. Inorg. Chem.* **2009**, *2009*, 1832–1843.

[3] K. Klauke, I. Gruber, T.-O. Knedel, L. Schmolke, J. Barthel, H. Breitzke, G. Buntkowsky, C. Janiak, *Organometallics* **2018**, *37*, 298–308.
[4] M. Grusch, *Exp. Hematol.* **2001**, *29*, 623–632.
[5] L. Messori, L. Marchetti, L. Massai, F. Scaletti, A. Guerri, I. Landini, S. Nobili, G. Perrone, E. Mini, P. Leoni, et al., *Inorg. Chem.* **2014**, *53*, 2396–2403.
[6] M. Baron, C. Tubaro, M. Basato, A. A. Isse, A. Gennaro, L. Cavallo, C. Graiff, A. Dolmella, L. Falivene, L. Caporaso, *Chem. - A Eur. J.* **2016**, *22*, 10211–10224.
[7] W. Kabsch, *Acta Crystallogr. Sect. D Biol. Crystallogr.* **2010**, *66*, 125–132.
[8] M. C. Burla, R. Caliandro, B. Carrozzini, G. L. Cascarano, C. Cuocci, C. Giacovazzo, M. Mallamo, A. Mazzone, G. Polidori, *J. Appl. Crystallogr.* **2015**, *48*, 306–309.
[9] G. M. Sheldrick, *Acta Crystallogr. Sect. A Found. Crystallogr.* **2008**, *64*, 112–122.
[10] P. Emsley, K. Cowtan, *Acta Crystallogr. Sect. D Biol. Crystallogr.* **2004**, *60*, 2126–2132.
[11] M. J. Frisch, G. W. Trucks, H. B. Schlegel, G. E. Scuseria, M. A. Robb, J. R. Cheeseman, G. Scalmani, V. Barone, G. A. Petersson, H. Nakatsuji, et al., **2016**.
[12] J. P. Perdew, K. Burke, M. Ernzerhof, *Phys. Rev. Lett.* **1996**, *77*, 3865–3868.
[13] W. J. Stevens, H. Basch, M. Krauss, *J. Chem. Phys.* **1984**, *81*, 6026–6033.
[14] M. Cossi, N. Rega, G. Scalmani, V. Barone, *J. Comput. Chem.* **2003**, *24*, 669–681.
[15] L. Galvez, M. Rusz, M. Schwaiger-Haber, Y. El Abiead, G. Hermann, U. Jungwirth, W. Berger, B. K. Keppler, M. A. Jakupec, G. Koellensperger, *Metallomics* **2019**, *11*, 1716–1728.
[16] O. Domarco, C. Kieler, C. Pirker, C. Dinhof, B. Englinger, J. M. Reisecker, G. Timelthaler, M. D. García, C. Peinador, B. K. Keppler, et al., *Angew. Chemie* **2019**, *131*, 8091–8096.
[17] C. Pirker, D. Lötsch, S. Spiegl-Kreinecker, F. Jantscher, H. Sutterlüty, M. Micksche, M. Grusch, W. Berger, *Exp. Dermatol.* **2010**, *19*, 1040–1047.
